# Conformational Entropy of Intrinsically Disordered Proteins from Amino Acid Triads

**DOI:** 10.1038/srep11740

**Published:** 2015-07-03

**Authors:** Anupaul Baruah, Pooja Rani, Parbati Biswas

**Affiliations:** 1 Department of Chemistry, University of Delhi, Delhi-110007, India

## Abstract

This work quantitatively characterizes intrinsic disorder in proteins in terms of sequence composition and backbone conformational entropy. Analysis of the normalized relative composition of the amino acid triads highlights a distinct boundary between globular and disordered proteins. The conformational entropy is calculated from the dihedral angles of the middle amino acid in the amino acid triad for the conformational ensemble of the globular, partially and completely disordered proteins relative to the non-redundant database. Both Monte Carlo (MC) and Molecular Dynamics (MD) simulations are used to characterize the conformational ensemble of the representative proteins of each group. The results show that the globular proteins span approximately half of the allowed conformational states in the Ramachandran space, while the amino acid triads in disordered proteins sample the entire range of the allowed dihedral angle space following Flory’s isolated-pair hypothesis. Therefore, only the sequence information in terms of the relative amino acid triad composition may be sufficient to predict protein disorder and the backbone conformational entropy, even in the absence of well-defined structure. The predicted entropies are found to agree with those calculated using mutual information expansion and the histogram method.

Conformational entropy of proteins is a proxy measure of its internal dynamics, which may be characterized by enumerating the different microscopic structural states involved in atomic motion^1^,^2^,^3^. Backbone conformational entropy constitutes a critical component of protein stability and plays a key role in the energetics of protein folding. Experimental measurement of conformational entropy has been difficult even though the atomic motions in the range of ps-ns may be characterized via NMR relaxation methods^4^, or from experiments like AFM-unfolding^5^ and neutron spectroscopy which demonstrates the role of conformational entropy in thermal protein unfolding^6^. Theoretical studies^7^,^8^ to quantify changes in conformational entropy are however computationally demanding. The first attempt, in this context^9^, calculated the relative entropy of protein unfolding by various residues with respect to glycine. A different approach^10^ correlated the backbone entropy to the distribution of main chain ϕ - ψ dihedral angles in the crystallographic structures by studying unfolded or flexible denatured states of proteins for a set of 61 nonhomologous proteins.

The conformational entropy of a protein may be characterized in terms of dihedral angles. Neglecting the time-dependent correlation of the dihedral angles may often lead to an upper bound of the value of conformational entropy^11^,^12^. The correlation between the dihedral angles may be extracted from molecular dynamics simulation trajectories coupled with the experimental NMR relaxation data for proteins through generalized order parameters, S^2^, derived from spin relaxation^13^,^14^. This method is not useful for the disordered proteins since they lack a well-defined structure. The computational approaches link atomic motions with conformational entropy through the principal component analysis of the variance-covariance matrix of protein’s internal or position co-ordinates. The eigenvalues of the variance-covariance matrix are used to evaluate the quasi-harmonic entropy via the entropy equation of the quantum mechanical harmonic oscillator^15^,^16^. This method overestimates the conformational entropy as the internal coordinates are assumed to be multidimensional Gaussian^17^,^18^,^19^, which may not be valid for the dihedral angles of disordered proteins. This assumption is hardly true for globular proteins where some dihedral angles follow non-Gaussian distribution. The mutual information expansion method^12^ evaluates the entropy by approximating this correlation between internal co-ordinates up to a certain order. However, this method is complicated by the sampling statistics and convergence problems at or beyond third order correlation, even for medium sized proteins^20^. A recent study by Genheden, Akke and Ryde^21^ infer that even long MD simulations do not completely equilibrate protein conformations, while the configurational entropy depends on both sampling statistics and simulation time. Hence, these methods have limited scope for the intrinsically disordered proteins (IDPs), where the disordered regions/domains are characterized by a conspicuous absence of interpretable electron density due to the fast motions of the atoms, rendering them invisible.

Intrinsically disordered proteins may be best represented as a dynamic ensemble of rapidly interconverting conformations^22^ either at the level of secondary or tertiary structures, resembling the unfolded protein under physiological conditions. In this context, Molecular Dynamics (MD) and Monte Carlo (MC) simulations are important in modeling conformational ensembles of IDPs/IDPRs. Despite the absence of a unique three-dimensional structure, disordered proteins exhibit functional diversity which complements the functions of ordered protein regions^23^,^24^,^25^. Another important feature of the intrinsically disordered proteins is their disorder-order transition caused by binding to specific targets which explains the mechanism of regulating various cellular processes like transcription, translation and cell cycle control^26^,^27^,^28^,^29^,^30^. Sequence analysis reveals that disordered domains/proteins are associated with less sequence complexity^31^. The sequences of these proteins are characterized by a low content of hydrophobic residues and a large number of charged residues which disfavor the folding process^26^,^32^ resulting in a lesser number of two-body contacts^33^.

This article quantifies the structural disorder of partially/completely disordered proteins in terms of both sequence composition and backbone conformational entropy, relative to that of the globular proteins. The non-redundant database along with the selected data sets of globular proteins, partially and completely disordered proteins (IDPRs and IDPs) are compiled separately for sequence analysis. MC and MD simulations characterize the conformational ensemble of the representative proteins of each group. A sequence analysis of the normalized relative composition for each individual amino acid reveals a considerable overlap between globular proteins and IDPs. However, the normalized relative composition calculated using amino acid triads is higher for IDPs as compared to the globular proteins and well demarcated from the region occupied by globular proteins. The conformational entropy of a disordered protein sequence may be expressed as the sum of the conformational entropy of the corresponding triads present in the non-redundant database obeying Flory’s isolated-pair hypothesis. Thus protein disorder may be predicted from the relative sequence composition only, while the backbone conformational entropy provides an appropriate measure of this structural disorder. The advantage of this method is that it avoids the requirement of extra long simulations which is not only computationally expensive but also time consuming.

## Materials and Methods

### Database Selection for Sequence Analysis

#### Non-redundant database

The non-redundant database chosen for this study comprised of non-homologous protein chains with a sequence identity of ≤*25%* (Feb 2010 release of PDB-select 1992–2009^34^). Proteins with X-ray crystallographic structures of resolution ≤*3* Å and R-factor ≤*0.3* are selected from the Protein Data Bank (PDB)^35^. The compiled non-redundant database consists of *4316* chains from *4163* proteins with chain lengths ranging from *25* to *1015* residues.

#### Globular Proteins

A data set of the globular proteins is compiled from the RCSB PDB with X-ray crystallographic structures of resolution ≤*3* Å. All proteins comprise of only single chains without any missing residues (listed in REMARK 465 of PDB). A sequence similarity of ≤*25%* and length ≥*40* residues is applied using PISCES server^36^. The final data set of globular proteins consists of *1917* chains with chain lengths ranging between *40* and *1724* residues.

#### Group I

A data set of protein chains with intrinsically disordered protein regions (IDPRs) is selected from the PDB. The disordered regions are characterized by missing residues in the electron density map of the respective X-ray crystallographic structures. A non-redundant data set of *9508* protein chains is obtained from the compiled database using PISCES server with the selection criteria of ≤*25%* and length ≥*40* residues. Out of these proteins, *138* chains with >*50%* structural disorder (percentage structural disorder is calculated with respect to the total length of the protein chain) comprise of the Group I data set. The chain lengths of these proteins span between *40* to *926* residues.

#### Group II

A set of *109* Intrinsically Disordered Proteins (IDPs) is selected from the DisProt, i.e., Database of Protein Disorder (Release 6.02)^37^. These proteins are completely disordered. A cutoff sequence similarity of ≤*25%* and length ≥*40* residues is applied on these proteins using PISCES server. The final data set of IDPs comprises of *91* proteins with chain lengths ranging between *40* and *1861* residues. The detailed method for the selection of globular, Group I and Group II proteins may be found in Ref. 38. The protein ID’s for the selected data sets of globular, Group I and Group II proteins are provided in the Supplementary information.

### Selected Proteins for Simulation

Conformational ensembles of the representative proteins from different groups are generated independently via Molecular Dynamics and Monte Carlo simulations. The representative globular protein is α-lactalbumin, whose crystal structure is extracted from the PDB (ID: *1A4V*). Disordered proteins are selected from two classes: (i) *1CD3, 1F0R* and *1MVF*, with well-defined secondary structure coexisting with highly flexible disordered regions (ii) α-synuclein, which lacks well-defined tertiary structures and is completely disordered. The representative proteins are selected because of the following reasons: (i) The selected proteins collectively possess the complete set of *20* amino acids in their disordered regions, (ii) All proteins exhibit varying degree of structural disorder with *1CD3* having the minimum percentage of disorder content and α-synuclein with maximum disorder content, (iii) Selected proteins have varying location of disordered regions i.e. at C-terminus of the sequence for 1MVF, N-terminus of the sequence for *1F0R*, in middle regions of the sequence for *1CD3* and throughout the entire sequence for α-synuclein. The three dimensional structures of the ordered regions of *1CD3, 1F0R* and *1MVF* are obtained from the PDB, while missing residues in the disordered regions are modeled with MODELLER^39^ using inputs from the protein sequence and structure of the ordered regions. The missing residues are incorporated by MODELLER such that the structure of the ordered part of the protein is exactly conserved. For α-synuclein, the sequence is the only input to model its structure. Supplementary Table S1 online summarizes the respective proteins used for MD and MC simulations with the method of modeling their disordered regions.

### Molecular Dynamics Simulation

Molecular Dynamics simulations are performed for each of the representative proteins (*1A4V, 1CD3, 1F0R, 1MVF* and α-synuclein) in explicit-water using AMBER 12 simulation package^40^. LEAP subroutine is used to add the missing hydrogen atoms in each of the protein structures. ff99SB force field with periodic boundary conditions for proteins and the TIP3P model^41^ for water is employed in the present study. This force field presents a careful reparametrization of the backbone torsion terms compared to ff99 and provides an improved proportion of helical versus extended structures^42^,^43^. Each protein structure is solvated in a cubic box (filled with TIP3P water) whose edge is maintained at a distance of *10* Å from the protein surface with closeness parameter *1* Å. The charge of each protein is neutralized by adding either Na+ or Cl– depending upon the charge of the solvated proteins. The PME algorithm^44^ with a real space cutoff of *8.0* Å is used for treating the long-range electrostatic interactions and a *8* Å distance cutoff is applied for the non-bonded interactions. The hydrogen atoms are constrained to the equilibrium bond length using the SHAKE algorithm^45^ which allows simulations with larger time step of *0.002* ps. Constant temperature and pressure are controlled through Berendsen’s temperature bath with coupling constant of *2* ps and barostat with a coupling constant of *1* ps, respectively^46^. Solvated proteins are energy minimized twice, first by energy minimization of the solvent, while keeping the protein constrained using conjugate gradient method followed by the energy minimization of the solvated protein. The minimized protein is initially equilibrated at an initial temperature of 100 K in NVT ensemble followed by gradually increasing the temperature upto 300 K at constant volume. A NPT equilibration of *5* ns is then performed for each solvated protein at a constant temperature of 300 K and a pressure of *1* bar followed by an extended NPT simulation of *100* ns. The root-mean-square deviation (RMSD) and radius of gyration (R_g_) are plotted as a function of the simulation time for each protein in Supplementary Fig. S1 online.

### Monte Carlo Simulation

Metropolis Monte Carlo simulation is also performed for *1CD3, 1F0R, 1MVF* and α-synuclein to complement the results of the Molecular Dynamics simulations. The *Cα* backbone of the modeled conformations of each of these four proteins are considered as input structures for the Monte Carlo simulations. The pseudo *Cα(i)-Cα(i* + *1)* and *Cα(i)-Cα(i* + *2)* bond lengths are restricted to *3.8* ± *0.15* Å^47^,^48^ and *6.0* ± *1.5* *Å*^49^ respectively. A 6–12 Lennard Jones potential accounts for the van der Waals interactions. The hydrophobic, electrostatic and the steric interactions are also modeled through coarse-grained two-body interaction potential. The energy function may be expressed as




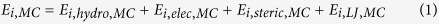




where,




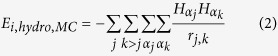




where, ***H***_***_α_j***_ and ***H***_***_α_k***_ are the normalized hydrophobicities of residues ***α***_***j***_ and ***α***_***k***_ respectively. ***r***_***j,k***_ is the distance between the sites j and k. The choice of the potential ensures that the hydrophobic-hydrophobic interactions are preferred while polar-polar interactions are not.




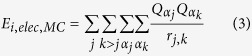







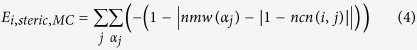




where, ***Q***_***_α_j***_ and ***Q***_***_α_k***_ are the charges on the residues *****α*****_***j***_ and *****α*****_***k***_ respectively. ***nmw***(*****α*****_***j***_) and ***ncn***(***i, j***) represents the normalized molecular weight of *****α*****_***j***_ and the normalized coordination number of the *j*^*th*^ site in the *i*^*th*^ conformation respectively. The steric energy function is chosen such that the residues with high molecular weight is preferred at sites with low coordination number, while the residues with low molecular weight are preferred at highly coordinated sites. For each Monte Carlo simulation, *2000* conformations are randomly selected for each of the representative proteins. These conformations for each protein are solvated using TIP3P water model and energy minimized using AMBER 12. Root-mean-square deviation (RMSD) and radius of gyration (R_g_) for the selected MC conformations are plotted for each protein in Supplementary Fig. S2 online.

### Residuewise Conformational Entropy

Backbone conformational entropy may be evaluated from the probability distribution of the ϕ - ψ dihedral angles of the main chain of polypeptides^10^,^50^. This entropy measure may be used as a criteria to distinguish the globular proteins from the disordered proteins, which lack well-defined secondary or tertiary structures. In this work, conformational entropy is calculated in terms of Shannon entropy which may be expressed as^8^,^51^,




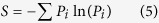




where ***P***_***i***_ is the fraction of amino acids present in the *i*^*th*^ bin for a specific range of ϕ - ψ angles of a given peptide segment. For each amino acid, the distribution of dihedral angles is obtained from the database of conformations. For the calculation of residuewise conformational entropy, individual amino acids are binned across the specified range of ϕ - ψ angles^8^. The Ramachandran’s plot is divided into *90* × *90* equally spaced grids; the height and width of each grid is 4°. Each amino acid in the protein is classified in a specific grid according to the values of its ϕ - ψ angles. The corresponding ***P***_***i***_ value is calculated from the fraction of the respective amino acid in that specific grid in the database. The conformational entropy of a protein is calculated from its ***P***_***i***_ values as defined in eq 5.

### Two-body Contacts

Average number of two-body contacts is a measure of the residue-residue interactions present in any protein which discriminates the globular proteins from the disordered ones. A pair of non-hydrogen atoms is considered to be in contact when they are separated by a distance less than *8* Å^52^. For any *i*^*th*^ residue of a protein, neighbors along the sequence (*i* *−* *1, i* *−* *2, i* + *1* and *i* + *2*) are neglected since contacts formed between these residues are present in the denatured state also with high probability^53^.

## Results and Discussions

The residuewise conformational entropy is calculated using eq 5 for each amino acid in the non-redundant database and the conformational ensembles of each of the representative proteins generated by MD and MC simulations. Conformational entropy of IDPs and IDPRs calculated using the conformational ensembles of proteins generated by MD simulations is compared with that in the non-redundant database and globular protein (*1A4V*) in Supplementary Fig. S3(a) online, while the same is depicted in Supplementary Fig. S3(b) online for the conformational ensembles generated by MC simulations.

**Figure 1:**
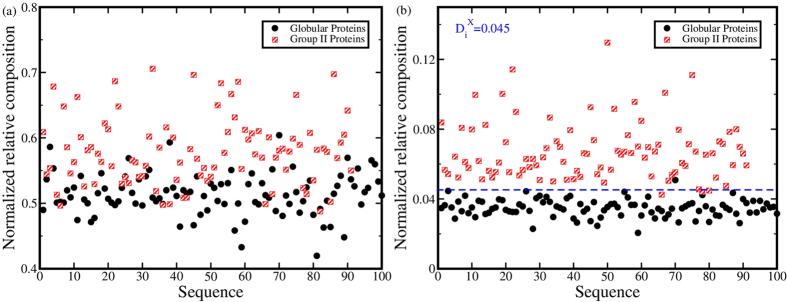
Normalized relative composition calculated using (**a**) individual amino acid, (**b**) triads of amino acids, as a function of sequences in data set of globular and Group II proteins. Blue line in (**b**) represents the boundary between globular and Group II proteins.

Relative composition of the individual amino acids is calculated from the fraction of that amino acid present in the data set of Group II proteins relative to that of the globular proteins. The relative composition of amino acid is given by




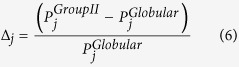




where j = *1* to *20*, ***P***_***i***_ = 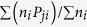, *P*_*ji*_ denotes the fraction of *j*^*th*^ residue in *i*^*th*^ sequence of length *n*_*i*_ and the summation is over all sequences in the respective data sets of proteins. The normalized relative composition is used to differentiate between the globular and Group II proteins which may be expressed as




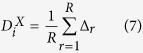




where *R* is the total number of residues in a sequence, Δ_*r*_ represents the normalized relative composition of *r*^*th*^ residue in a sequence in the data set *X* for either globular proteins or Group II proteins. 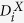 is calculated for individual sequences in the data set of globular and Group II proteins and the values are plotted in Fig. 1(a). The figure depicts a fuzzy boundary between the globular and Group II proteins, even though Group II proteins, on an average, show a higher value of the normalized relative composition. Thus, a new method is proposed which considers the combination of three amino acids, rather than the individual ones, to distinguish the Group II proteins from those of the globular ones. The combination of three amino acids, termed as amino acid triads (total combination = *8000*) is considered, since the conformational flexibility of a specific amino acid residue is dependent on its flanking neighbors at the two ends. The normalized relative composition of these triads are calculated for each sequence of the globular and Group II proteins. The calculation of the normalized relative composition of the triad is similar to that of the individual amino acids using eqs 6 and 7. The normalized relative composition is calculated for all possible triads (i.e. *20*^3^ *=* *8000*). For amino acid triads, *P*_*ji*_ is the fraction of the *j*^*th*^ triad in the *i*^*th*^ sequence and *R* represents the total number of such triads in the sequences of the data sets for globular and Group II proteins. It is found that triads *HHH*, *LEH*, *ALS* and *VLD* are least preferred (except the triads which do not exist in Group II proteins) while triads *QPF, PQQ, PHW* and *QQP* are highly preferred in Group II proteins (see Supplementary Fig. S4 online). In Supplementary Fig. S4 (a) the relative composition of the top *50* triads, which are most preferred in Group II proteins, are plotted with *99%* confidence interval for *2000* bootstrap resampling iterations. Supplementary Fig. S4 (b) depicts the relative composition of *50* triads which do not exhibit any clear preference; increased or decreased frequency of these triads in any sequence occurs by chance. The relative composition of the *50* least preferred triads are plotted in Supplementary Fig. S4 (c) online. In Fig. 1(b), 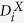 is plotted for individual sequence in the data set of globular and Group II proteins. This figure depicts a higher value of the normalized relative composition for Group II proteins with a distinct boundary separating the Group II proteins from the globular proteins. Thus, the composition of amino acid triads provides a novel method to differentiate between the disordered and globular proteins and hence serve as an appropriate yardstick to predict disorder. The distribution of the normalized relative composition calculated using amino acid triads is plotted for data set of globular, Group I and Group II proteins (see Supplementary Fig. S5 online). The results show that the disordered proteins may be differentiated from well-structured globular proteins from the sequence information only.

**Figure 2:**
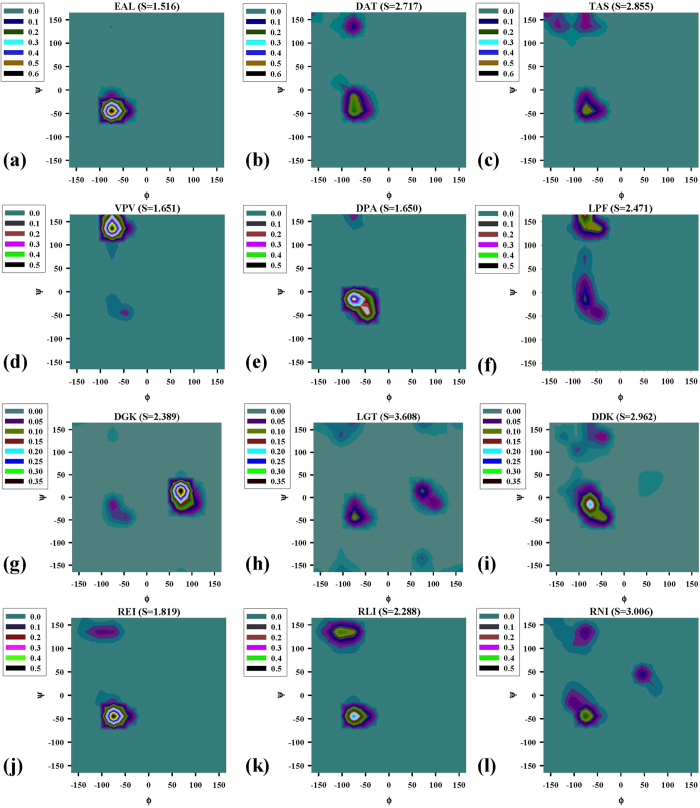
The probability distribution of ϕ - ψ angles in the non-redundant database for the following triads: (**a**) EAL, (**b**) DAT, (**c**) TAS, (**d**) VPV, (**e**) DPA, (**f**) LPF, (**g**) DGK, (**h**) LGT, (**i**) DDK, (**j**) REI, (**k**) RLI and (**l**) RNI. The corresponding entropy values are mentioned in parentheses.

**Figure 3:**
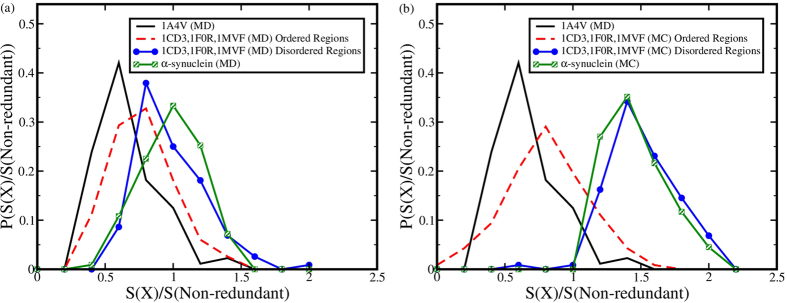
Conformational entropy calculated using triads relative to non-redundant database for (**a**) Molecular dynamics, (**b**) Monte Carlo generated ensembles of conformations.

The above results confirm that the choice of amino acid triads provides a simple and effective method to predict disorder in proteins. This may suggest that these triads encode important information about the stability, flexibility and conformational entropy of proteins. Thus, the conformational entropy of these triads are calculated depending on the distribution of ϕ - ψ angles for different conformational ensembles of the non-redundant database, globular (*1A4V* ) protein, IDPRs (*1CD3, 1MVF, 1F0R*), and IDPs (α-synuclein) respectively. The conformational entropy of each conformational ensemble may be calculated by the histogram method as




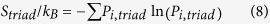




where *P*_*i,triad*_ is the fraction of triads present in the *i*^*th*^ bin for a given range of the dihedral angles, ϕ - ψ. The Ramachandran plot may be divided into *12* × *12* equally spaced grids with height and width of 30° for each grid. A sufficiently large bin size i.e. 30° is chosen to minimize the statistical errors in the calculated probabilities of amino acid triads. Each amino acid triad in the protein is binned in the specified grid according to ϕ - ψ angles of middle amino acid in triad. The value of *P*_*i,triad*_ is calculated from the fraction of triads in the specific grid of the database. The probability distributions of the triads in the non-redundant dataset are depicted in Fig. 2. In Fig 2(a–c) the probability distribution contour is plotted for triads *EAL*, *DAT* and *TAS*. Despite identical middle residue in each of these triads, the residue Alanine populates different regions of the dihedral angle space in the Ramachandran plot. Among these three triads *EAL* has the least entropy (S = *1.516*) and is restricted to the α-helix region in the Ramachandran plot, *DAT* (S = *2.717*) populates the α-helix and PP-II helix region, while *TAS* (S = *2.855*) populates α-helix, PP-II helix and β-sheet regions. This suggests that the neighboring flanking residues of an amino acid may have significant influence on the structural degrees of freedom of that amino acid. Similarly, Proline, which is known to be structurally rigid, may exhibit varied structural flexibility and conformational entropy as is evident from Fig. 2(d–f). The highly flexible Glycine may contribute differently to the conformational entropy of a protein depending on its nearest neighbors in the triad as depicted in Fig. 2 (g,h). Interestingly, Aspartic acid is found to have more entropy as compared to Glycine, when it is flanked by residues *D* and *K* (Fig. 2 (i)). Figure 2 (j–l) depicts the change of the conformational entropy of the amino acid triads with the change in the middle amino acid residue only. Among these three triads, *REI* exhibits the lowest entropy (S = *1.819*) while *RNI* exhibits highest entropy (S = *3.006*). Supplementary Table S2 lists the relative entropies of amino acid triads (*X*_*1*_*X*_*2*_*X*_*3*_), which show higher entropy relative to *X*_*1*_*GX*_*3*_, with 99% confidence interval for 2000 bootstrap resampling iterations. The relative entropies of the triads that exhibit minimum entropy with respect to *X*_*1*_*GX*_*3*_ are given in Supplementary Table S3 online. Figure 2 and Supplementary Tables S2 and S3 imply that changing the middle amino acid for the same flanking residues as well as changing the flanking residues for the same middle amino acid may have significant impact on the conformational entropy. Therefore, study of triads in terms of conformational entropy is important. Conformational entropy of each amino acid triad is evaluated for globular (*1A4V*), IDPRs (*1CD3, 1F0R* and *1MVF*) and IDPs (α-synuclein) using MD and MC generated conformational ensembles. The ratio of the conformational entropy of a specific triad in a data set of proteins to the corresponding conformational entropy of the triad in non-redundant database is calculated and the probability distribution of this ratio is plotted in Fig. 3(a,b) for MD and MC simulations respectively. Globular proteins exhibit the least entropy value with an average of ~0.65, which implies that a triad present in a protein with well-defined structure actually populates approximately half of the accessible ϕ - ψ range allowed for that triad. The conformational entropy of the ordered regions of IDPRs are flanked between their disordered counterparts and the globular proteins. α-synuclein depicts the highest entropy value for both MD (with average entropy value of ~1.0) and MC (with average entropy value of ~1.47) generated conformational ensemble. This implies that in a completely disordered protein, an amino acid triad spans the entire range of allowed ϕ - ψ angles over a period of time, imparting high flexibility and consequently high conformational entropy to the structural ensemble.

The ratio of the conformational entropy of any triad in the conformational ensembles of the partially and completely disordered proteins to that in the non-redundant database is calculated for conformational ensembles generated by MD simulations at *60* ns, *80* ns and *100* ns. The probability distribution of this ratio is plotted in Supplementary Figs S8 and S9 for partially disordered and completely disordered proteins. Despite the slight difference in the conformational entropy values for the *60* ns and *100* ns MD trajectory, *80* ns and *100* ns MD trajectories depict a similar distribution of the conformational entropy for both partially and completely disordered proteins. The position of the maximum is coincident for both *80* ns and *100* ns MD trajectories in both figures. This implies a *100* ns MD simulation may be sufficient to sample the ϕ - ψ range of the triads in disordered proteins. Recent studies^54^,^55^,^56^ also support the fact that a *100* ns long MD simulation may adequately sample the dihedral angle space of the intrinsically disordered proteins. It is observed that dihedral angles are strongly correlated for IDPs, a few most populated dihedral combinations may dominate a major fraction of the entire conformational ensemble^55^. Therefore longer simulations may be helpful to sample more conformations which are not frequently populated but the overall ϕ - ψ distribution and the position of the maximum may not alter.

**Figure 4:**
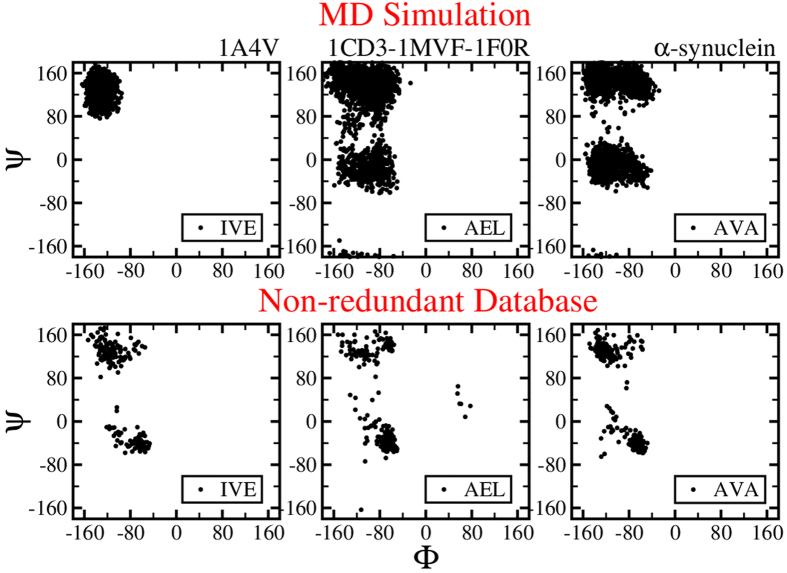
ϕ - ψ angles of the triad *IVE* in globular (*1A4V*), *AEL* in IDPRs (*1CD3*, 1*MVF* and *1F0R*) and *AVA* in IDP (α-synuclein). The ϕ - ψ angles are extracted from the MD simulation generated conformational ensemble and non-redundant database.

The ϕ - ψ angles of the selected triads from each ensemble of conformations of globular (*1A4V*), IDPRs (*1CD3, 1F0R* and *1MVF*) and IDPs (α-synuclein) are plotted in Fig. 4 (also see Supplementary Figs S6 and S7 online). These figures clearly demonstrate that the amino acid triads (*LLK, IVE* and *NKL*) in globular proteins span approximately half of the ϕ - ψ region as compared to the non-redundant data set of proteins. This may be due to the fact that triads in globular proteins exhibit a propensity for either helices or sheet structures and hence span limited regions of the Ramachandran space. While the amino acid triads in IDPRs (*LAE, AEL* and *TLA*) and IDPs (*AAA, AVA* and *AEK*) populate the entire range spanned by the non-redundant database of proteins. Since, disordered proteins lack specific secondary structures, triads in IDPRs and IDPs assume all possible ϕ - ψ angles in the allowed dihedral angle space. The triads in IDPRs and IDPs thus obey the Flory’s isolated-pair hypothesis^57^, which states that the ϕ - ψ angles for a given pair of residues is independent of those of the adjoining pair of residues (except for proline and residues preceding proline residues) in a protein. Within this approximation, we propose that the conformational entropy of a disordered protein sequence can be expressed as the sum of the conformational entropy of the corresponding triads present in the non-redundant database.




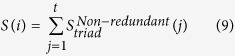




where *S(i)* represents the conformational entropy of the *i*^*th*^ disordered protein, *t* is the total number of triads in *i*^*th*^ protein sequence and 
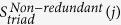
 denotes the entropy of *j*^*th*^ triad in the non-redundant database. Similarly for a sequence of a globular protein, the average conformational entropy may be estimated as




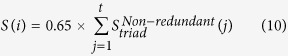




Mutual information expansion method is used to verify the validity of the results obtained from our entropy prediction method. Systematic expansion of entropy in mutual-information terms upto second order may be written as^12^




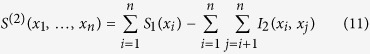




where




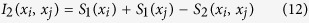




**Figure 5:**
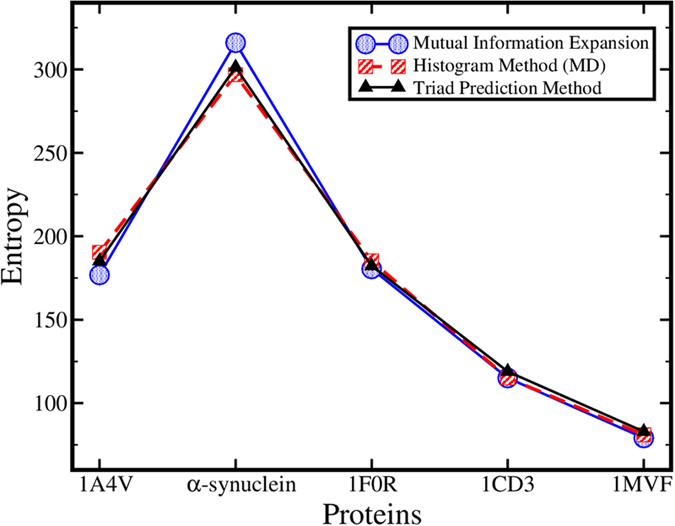
Conformational entropy calculated using mutual information expansion, histogram and our method (using eqns 9 and 10).

Here, *I*_*2*_(*x*_*i*_*,x*_*j*_), is a measure of the correlation between two degrees of freedom (dihedral angles in this case), which denotes the mutual information of the system. *S*_1_(*x*_*i*_) and *S*_1_(*x*_*j*_) represents the uncorrelated Shannon entropies for *x*_*i*_ and *x*_*j*_ dihedral angles respectively. *S*_*2*_(*x*_*i*_, *x*_*j*_) is the correlated entropy for the pair of dihedrals *x*_*i*_ and *x*_*j*_ . For *1A4V*, the correlation of *i*^*th*^ dihedral with *i* + *1, i* + *2* and *i* + *3* is found to be *0.07, 0.016* and *0.012* per dihedral pairs, respectively, for grids with height and width of 30°. The correlation between two dihedrals is found to decrease with an increase in the distance between dihedrals along the sequence. Thus, the correlation of each dihedral angle is calculated upto the nearest neighbor (i.e. for *i*^*th*^ dihedral, correlation is considered upto the *(i* + *1)*^*th*^ dihedral). The conformational entropy predicted using our method (using eqns *9* and *10*) is compared (see Fig. 5) with those calculated from the mutual information expansion method and simple histogram method that utilizes the MD generated conformational ensemble of each protein. Conformational entropy is calculated for *1A4V,* α-synuclein and disordered regions of *1F0R, 1CD3* and *1MVF*. Figure 5 depicts the match in the entropy value calculated using our method with those calculated using mutual information expansion (a maximum *5.7%* difference) and histogram binning method (a maximum *3.3%* difference). Hence, our sequence-based method provides fairly accurate estimation of the conformational entropy using the sequence information only, with the nearest neighbor pair correlation.

MC generated conformations for IDPRs and IDPs depict a wider range of ϕ - ψ angles compared to that of the non-redundant database. This is due to the choice of the coarse-grained potential for MC simulations with less restrictions imposed in the accessible conformations. Thus this study proposes a method to predict the disorder in proteins from the relative composition of the amino acid triads including a measure of an average conformational entropy for that sequence, since it may not be feasible to determine the conformational entropy of a large number protein sequences, especially for the completely disordered proteins, by simulations.

The lower conformational entropy for globular proteins may be attributed to the highest number of favorable two-body contacts which are the primary stabilizing factor for well-defined structures. The two-body contacts are calculated using non-redundant database and conformational ensembles generated from MD and MC simulations respectively (*1A4V*, *1CD3, 1F0R, 1MVF* and α-synuclein) with a distance cutoff of *8* Å. The distribution of two-body contacts for all data sets of proteins is plotted in Supplementary Fig. S10 online. The non-redundant database, comprising of well-structured globular proteins shows the highest number of contacts in their native state. Both IDPRs and IDPs depict less number of average two-body contacts in the conformational ensemble generated by MD simulation. For the MC generated conformational ensemble of *1CD3, 1F0R, 1MVF* and α-synuclein, the two-body contacts depict a broad distribution, with the peaks matching with respective ensembles of MD simulation. The broad distribution of MC simulation generated ensemble is due to use of the coarse-grained model with less restrictions.

## Conclusions

This work quantitatively characterizes the structural order/disorder in proteins in terms of the backbone conformational entropy, number of two-body contacts and the relative composition of amino acids compared to those of the globular proteins. Three groups, comprising of globular, Group I and Group II proteins, are compiled from the PDB and DisProt database for sequence analysis. MD and MC simulations are used to characterize the conformational ensemble of the representative proteins of each group. Sequence analysis of these groups of proteins reveal substantial overlap between the globular and completely disordered proteins from the normalized relative composition of individual amino acids. However, the normalized relative composition calculated using amino acid triads depicts a distinct boundary separating globular and disordered proteins. Thus the structural order/disorder in a protein may be accurately predicted from the sequence information only. The analysis of the conformational entropy of the triads is important. It is observed that a change in the middle amino acid of a triad or change in the neighboring flanking residues of a specific amino acid affects the conformational stability and flexibility of triads, which in turn may affect the conformational entropy of the protein. The conformational entropy evaluated from the heterogeneous conformational ensemble of the representative proteins from each group reveal that in globular proteins the amino acid triads samples about half of all possible conformational states while those in a disordered protein follow the Flory’s isolated-pair hypothesis and sample the entire range of allowed ϕ - ψ angles. Thus, conformational entropy of a disordered protein sequence may be expressed as the sum of the conformational entropy of the corresponding triads present in the non-redundant database. The conformational sampling and convergence of MD simulations for IDPs/IDPRs is inconclusive. Even microsecond long MD simulations sometimes do not exhibit complete convergence^58^. However, it is observed a *100* ns long MD simulation may capture the ϕ - ψ distribution effectively^54^,^55^. The predicted values of the conformational entropy for globular and disordered proteins agree well with those calculated using mutual information expansion and histogram method. Thus, our sequence-based method may estimate the conformational entropy of proteins upto nearest neighbor pair correlation without the need of computationally expensive and time consuming simulations. Higher conformational entropy of the intrinsically disordered proteins is also reflected in the less number of two-body contacts, which is the primary stabilizing factor for well-structured globular proteins. Thus protein disorder may be predicted from the relative sequence composition of amino acid triads only, while the backbone conformational entropy provides an appropriate measure of this structural disorder.

## Additional Information

**How to cite this article**: Baruah, A. *et al*. Conformational Entropy of Intrinsically Disordered Proteins from Amino Acid Triads. *Sci. Rep*. **5**, 11740; doi: 10.1038/srep11740 (2015).

## Supplementary Material

Supplementary Information
