# Supplementary material for: Conformational Entropy of Intrinsically Disordered Proteins from Amino Acid Triads
Source: Sci Rep. 2015 Jul 3;5:11740. doi: 10.1038/srep11740 (PMC4490338; doi:10.1038/srep11740)
Supplement: Supplementary Information [file srep11740-s1.pdf]

Supplementary Information

Conformational Entropy of Intrinsically Disordered Proteins from Amino Acid Triads

Anupaul Baruah, Pooja Rani and Parbati Biswas*
Department of Chemistry,
University of Delhi,
Delhi: 110007, India.
*Email: pbiswas@chemistry.du.ac.in

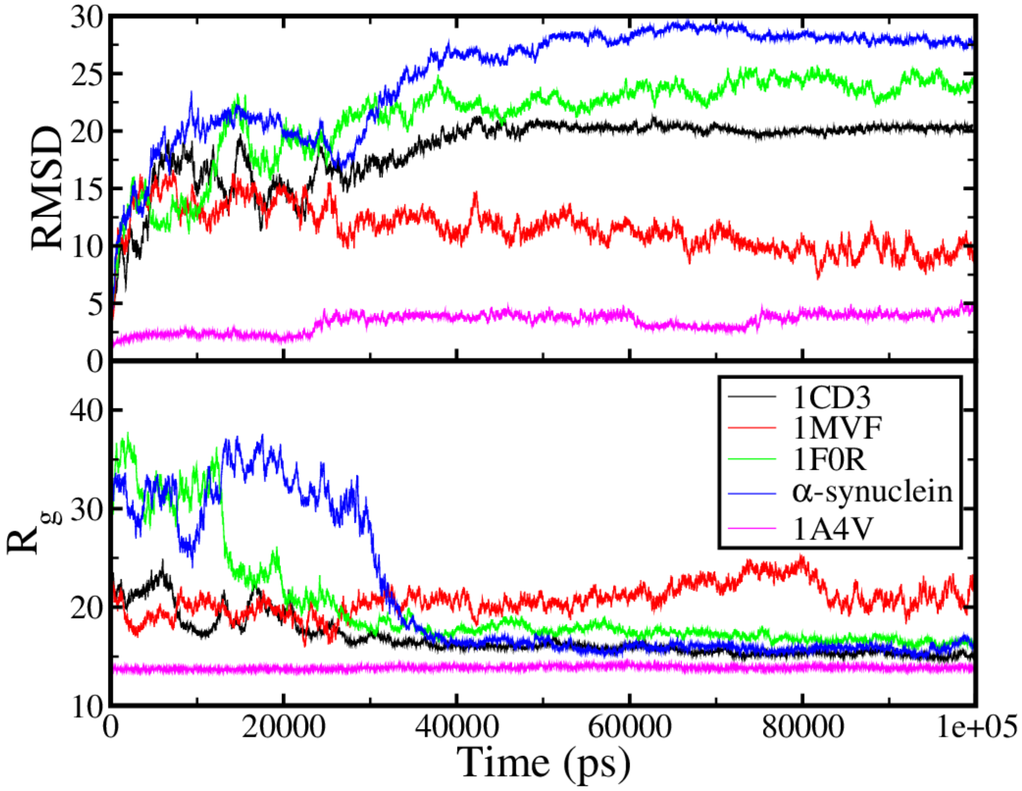

Supplementary Figure S1. RMSD (adapted with permission from DOI:10.1021/jp511961c. Copyright 2015 American Chemical Society.) and Radius of gyration (R_g) as a function of MD simulation time.

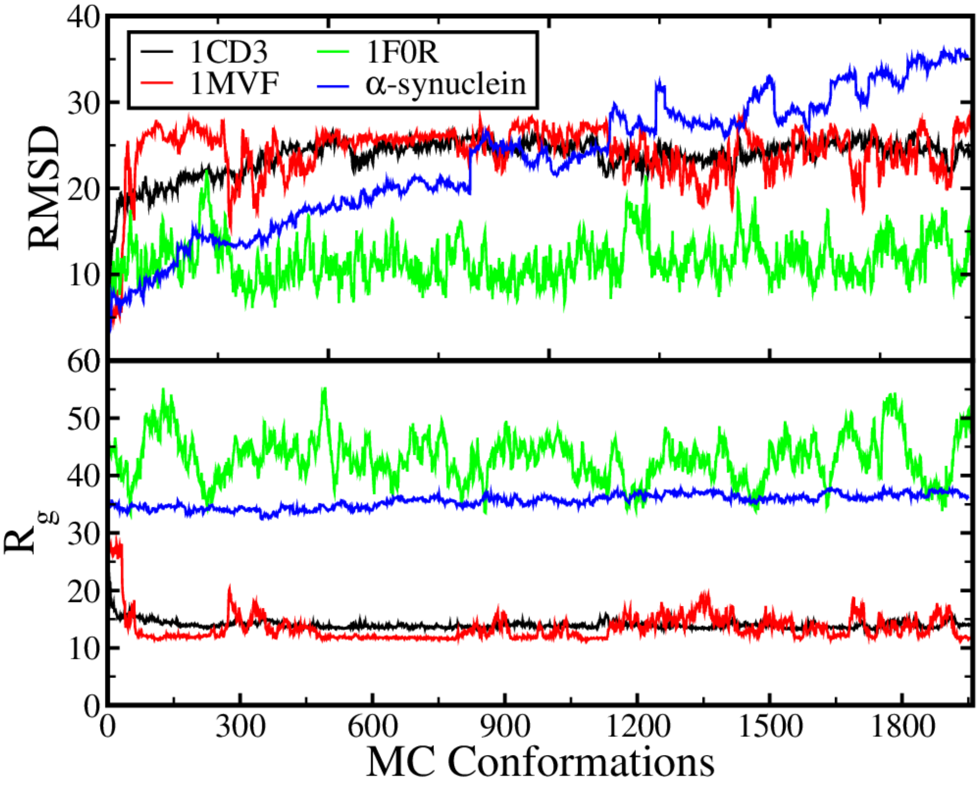

Supplementary Figure S2. RMSD and Radius of gyration (R_g) as a function of MC generated conformations.

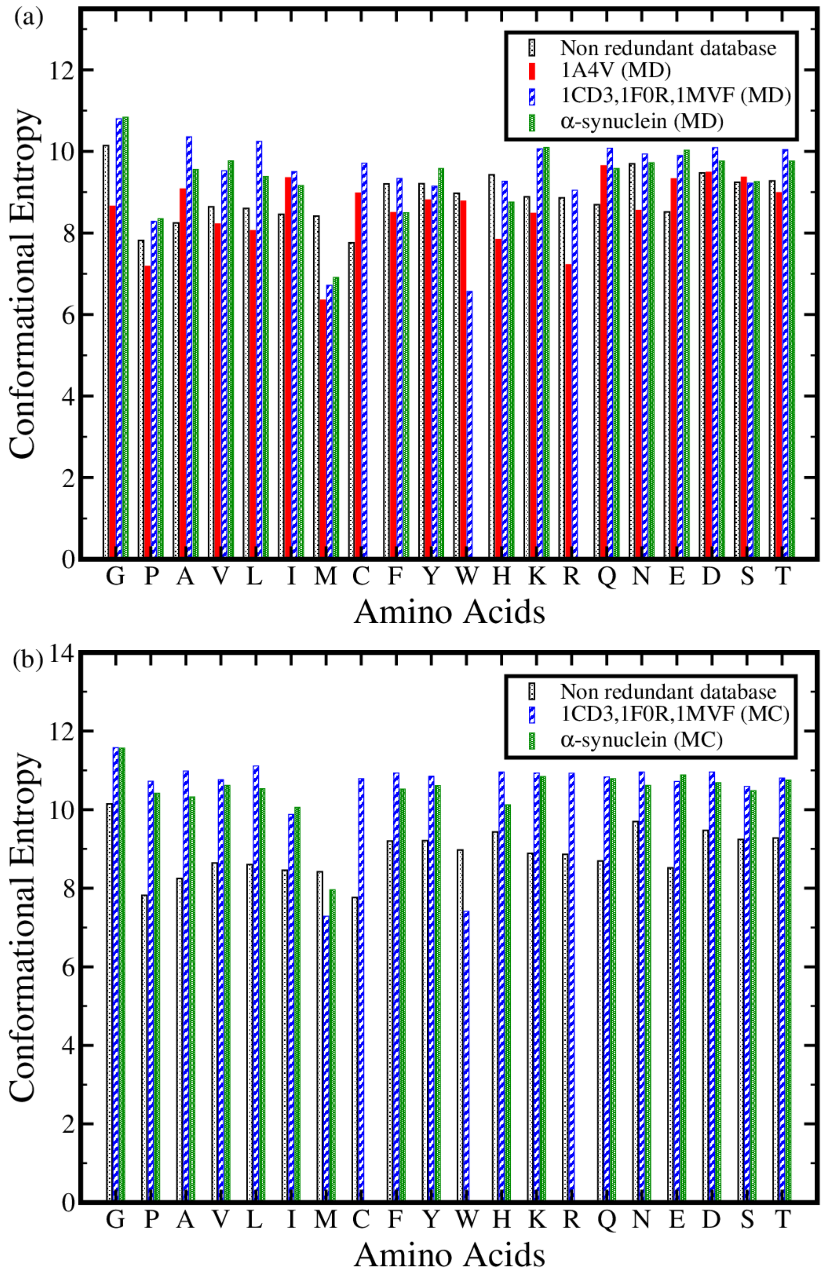

Supplementary Figure S3. Residuewise conformational entropy for ensembles of (a) Molecular dynamics, (b) Monte Carlo generated conformations.

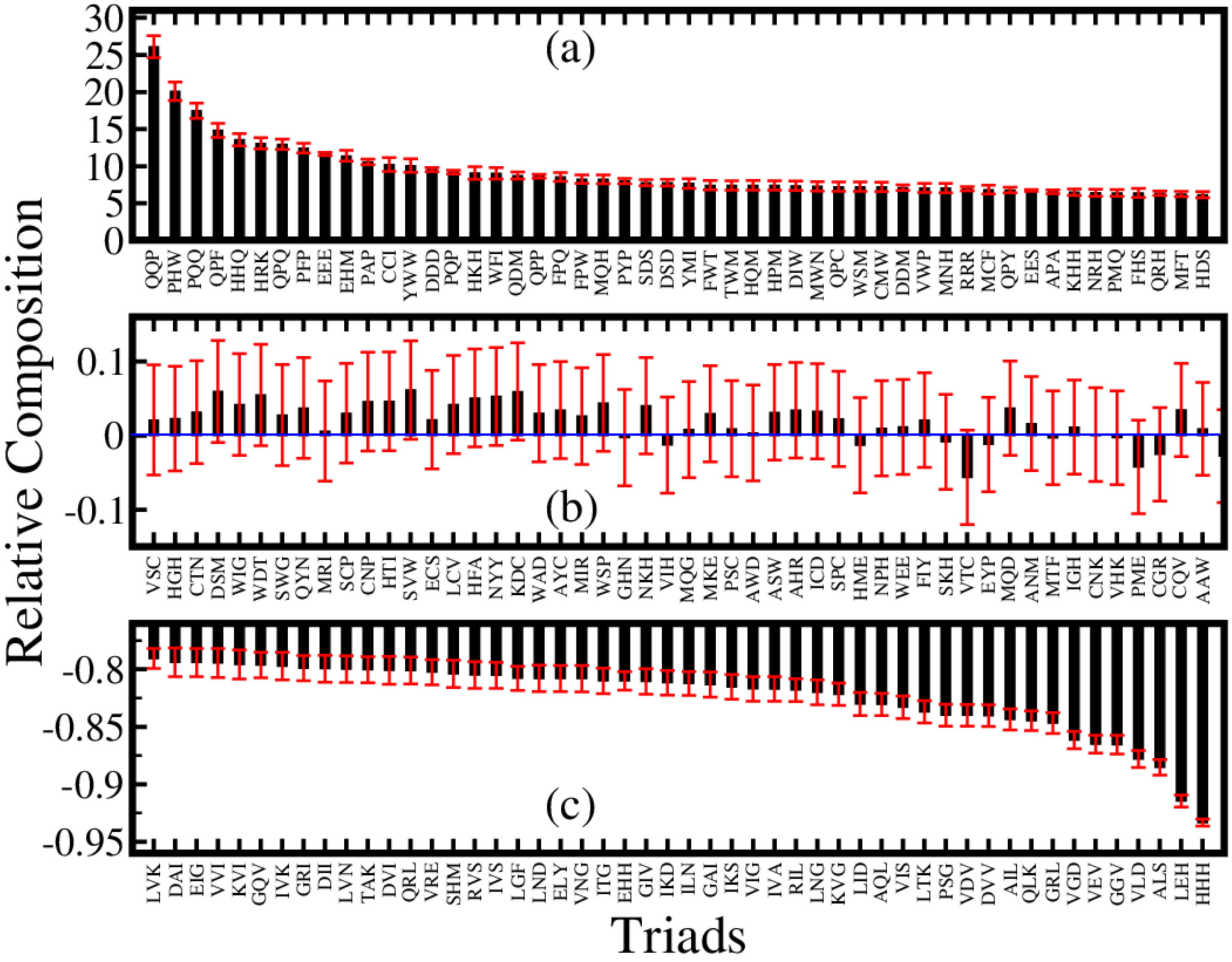

Supplementary Figure S4. The relative composition of (a) top 50 most preferred triads, (b) 50 triads with no clear preference and (c) 50 least preferred triads in Group II proteins w.r.t to the Globular proteins. 99% confidence intervals for 2000 bootstrap resampling iterations are also shown. Out of all possible 8000 triads, 2795 triads are not considered in this analysis of which 2756 triads do not exist only in Group II proteins, 2 triads do not exist only in Globular proteins and 37 triads are not present in both Group II and Globular proteins.

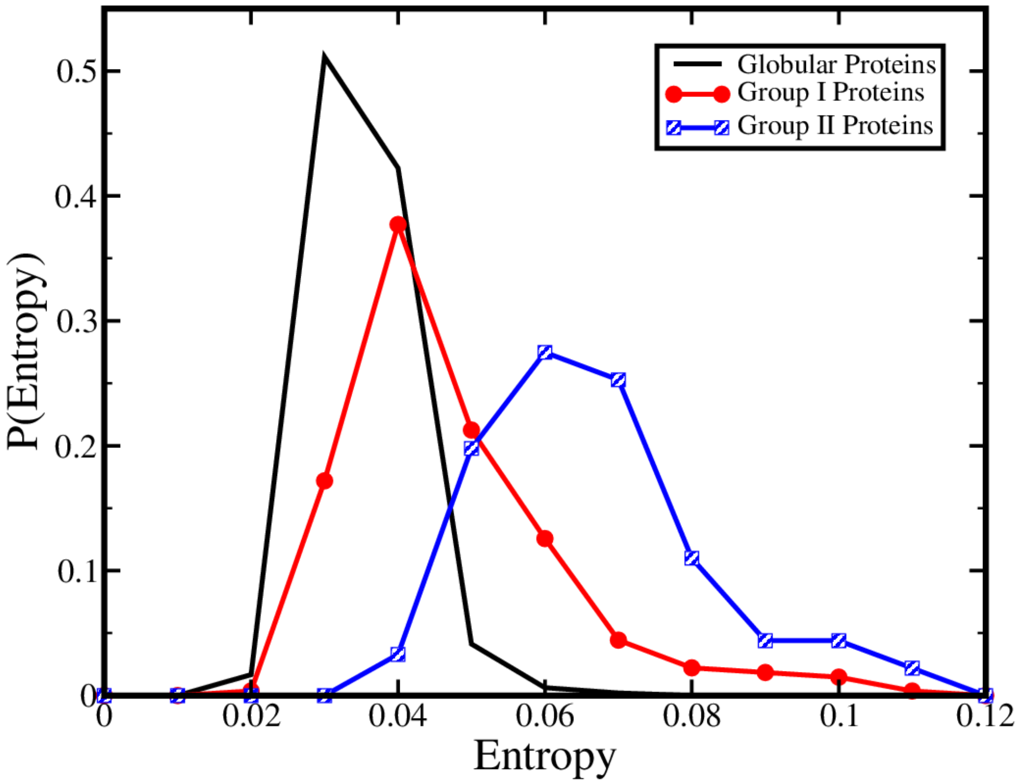

Supplementary Figure S5. Distribution of normalized relative composition calculated using triads of amino acid for data set of globular, Group I and Group II proteins. The globular proteins record the lowest normalized relative composition, while Group II proteins depict the highest value.

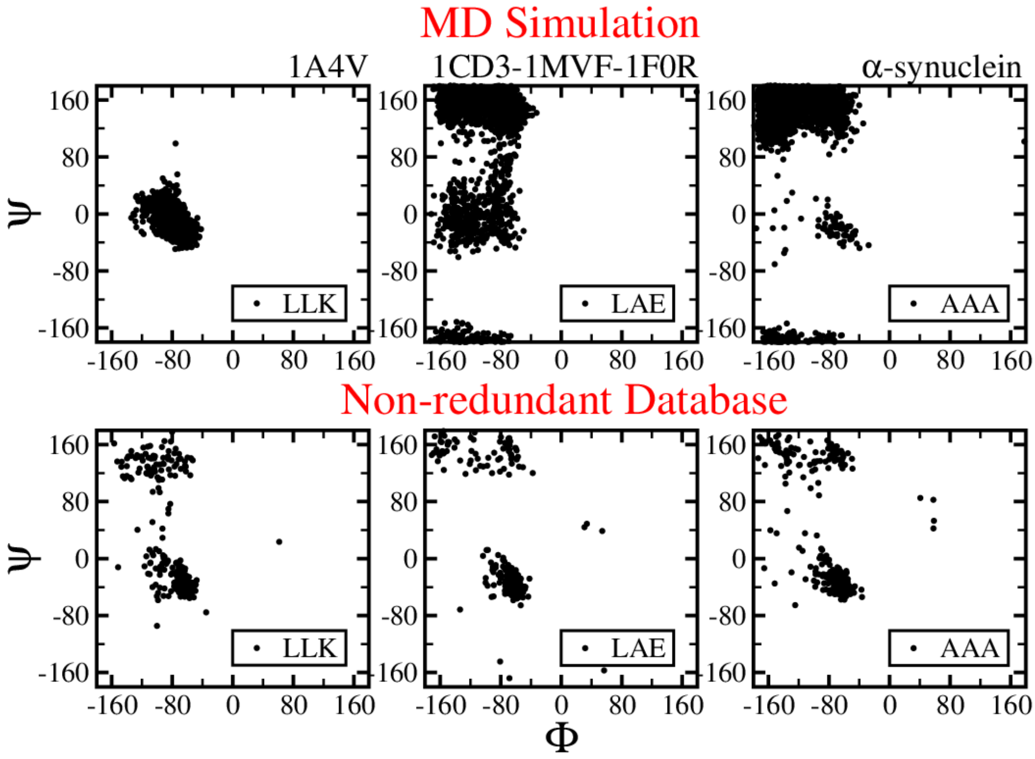

Supplementary Figure S6. $\phi - \psi$ angles of the triad LLK in globular ($1A4V$), LAE in IDPRs ($1CD3$, $1MVF$ and $1F0R$) and AAA in IDP (α-synuclein). The $\phi-\psi$ angles are extracted from the MD simulation generated conformational ensemble and non-redundant database.

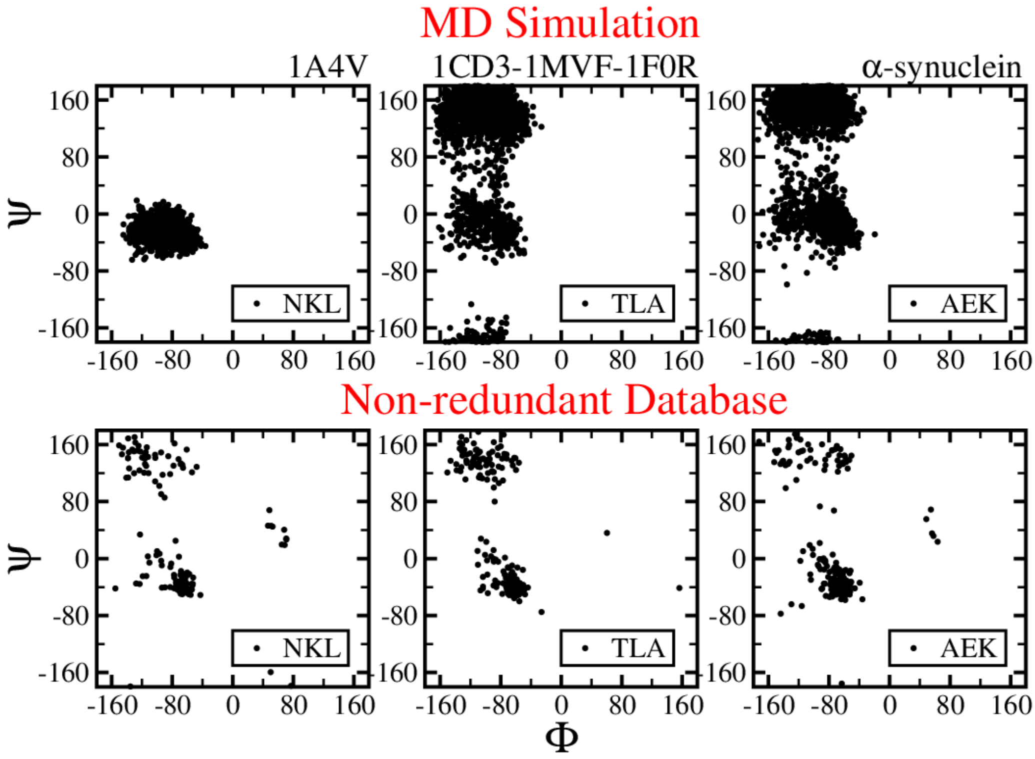

Supplementary Figure S7. $\phi - \psi$ angles of the triad NKL in globular ($1A4V$), TLA in IDPRs ($1CD3$, $1MVF$ and $1F0R$) and AEK in IDP (α-synuclein). The $\phi{-}\psi$ angles are extracted from the MD simulation generated conformational ensemble and non-redundant database.

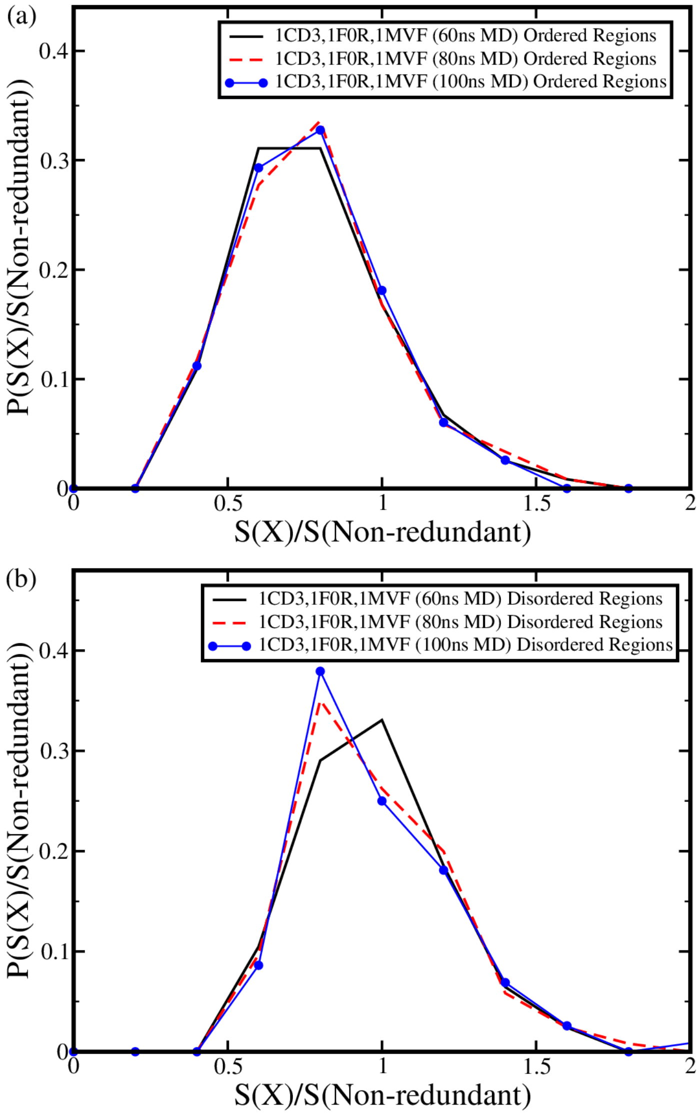

Supplementary Figure S8. Conformational entropy calculated using triads relative to non-redundant database for Molecular Dynamics generated ensembles of conformation for (a) ordered and (b) disordered regions of partially disordered proteins (1CD3, 1F0R and 1MVF).

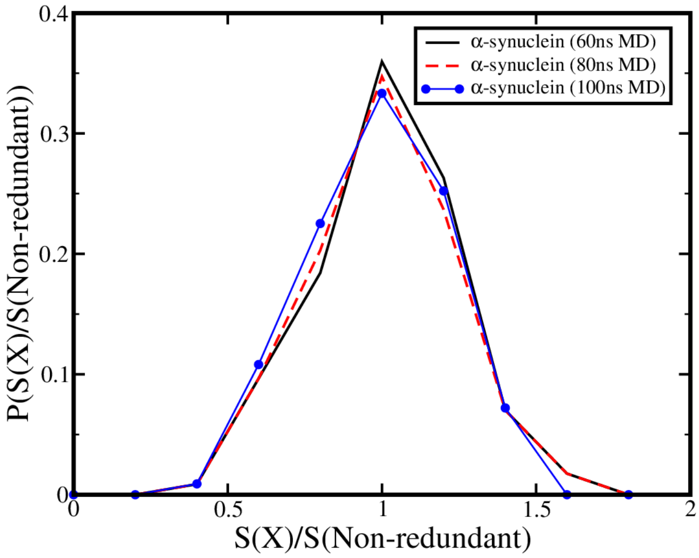

Supplementary Figure S9. Conformational entropy calculated using triads relative to non-redundant database for Molecular Dynamics generated ensembles of conformation for α-synuclein.

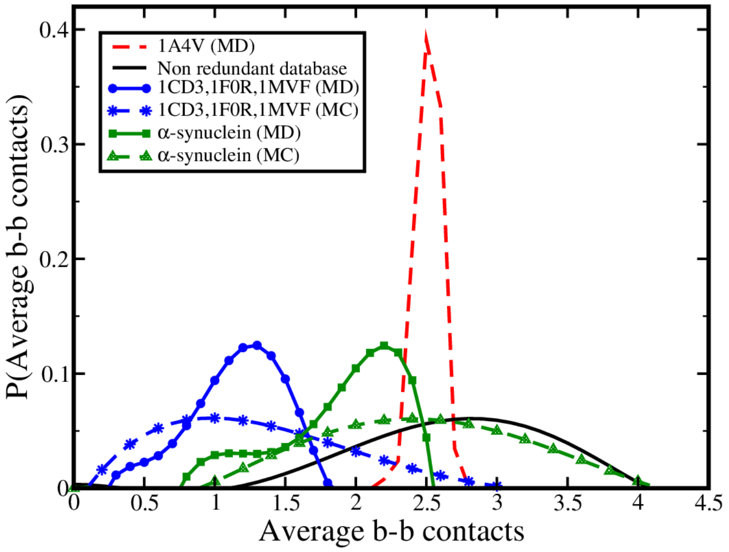

Supplementary Figure S10. Distribution of average native contacts.

Supplementary Table S1. Selected proteins for MD and MC simulations.

Protein	PDB ID	Ordered /Disordered	Disordered Residues (Total Residues)	Structure
α-lactalbumin	1A4V	Ordered	— (123)	Extracted from RCSB
Scaffolding Protein GPB	1CD3	Partially Disordered	9-60 (120)	Initial template is extracted from RCSB. Disordered region is modeled by MODELLER
PemI-like Protein	1MVF	Partially Disordered	1-3, 48-82 (82)	Initial template is extracted from RCSB. Disordered region is modeled by MODELLER
Human Factor Xa	1F0R	Partially Disordered	1-82 (134)	Initial template is extracted from RCSB. Disordered region is modeled by MODELLER
α-synuclein	—	Completely Disordered	1-140 (140)	Complete protein is modeled by MODELLER

Supplementary Table S2. Relative entropy ($=(S(X_1X_2X_3) - S(X_1GX_3))/S(X_1GX_3)$) of triads ($X_1X_2X_3$) w.r.t X_1GX_3 where entropy of a specific triad is found to be higher than the entropy of X_1GX_3.

Triads	Relative Entropy w.r.t X_1GX_3	Confidence Interval	Triads	Relative Entropy w.r.t X_1GX_3	Confidence Interval
WVM	0.48	0.157	WLM	0.33	0.278
HLM	0.31	0.012	HSM	0.28	0.013
WDM	0.25	0.282	PNC	0.24	0.011
HHM	0.24	0.021	HVM	0.23	0.012
HHH	0.23	0.005	DDK	0.21	0.004
DNK	0.21	0.004	PLC	0.21	0.012
NNR	0.20	0.005	PIC	0.17	0.011
DKK	0.16	0.003	TNC	0.16	0.012
PKC	0.16	0.011	TEC	0.16	0.011
FEW	0.16	0.008	NNK	0.15	0.004
NNQ	0.15	0.004	NDK	0.15	0.004
NDR	0.14	0.004	PDC	0.14	0.011
DNH	0.14	0.005	DYK	0.14	0.004
DNT	0.14	0.003	NNV	0.13	0.003
DFT	0.13	0.003	DFS	0.13	0.003
DNS	0.12	0.003	NDV	0.12	0.004
FDW	0.12	0.009	TAC	0.12	0.009
MDG	0.12	0.005	DFK	0.12	0.003
FAW	0.12	0.008	PFD	0.11	0.004
NLH	0.11	0.006	PVC	0.11	0.011
NSR	0.11	0.005	CSL	0.10	0.006
DKS	0.10	0.003	DDT	0.10	0.003
DYS	0.10	0.003	NTK	0.10	0.004
DSK	0.10	0.003	DDW	0.10	0.005
FSW	0.10	0.008	DSS	0.09	0.003
WLH	0.09	0.008	FNW	0.09	0.009
DTT	0.09	0.003	NYK	0.09	0.004
DIS	0.09	0.003	PLD	0.09	0.003
DRS	0.08	0.003	CDI	0.08	0.005
DYT	0.08	0.003	MNR	0.08	0.007
DLK	0.08	0.003	HDM	0.08	0.011
LSC	0.08	0.005	FDC	0.08	0.007
DNQ	0.08	0.004	WDI	0.08	0.006
DNR	0.08	0.004	HDY	0.08	0.006
CDL	0.07	0.005	TVC	0.07	0.010

Triads	Relative Entropy w.r.t X_1GX_3	Confidence Interval	Triads	Relative Entropy w.r.t X_1GX_3	Confidence Interval
PAC	0.07	0.011	VDM	0.07	0.004
DLT	0.07	0.003	CLP	0.07	0.008
ENQ	0.07	0.004	DST	0.07	0.003
NSK	0.07	0.004	TSC	0.07	0.010
DDR	0.06	0.004	DIT	0.06	0.003
PNE	0.06	0.003	DYH	0.06	0.005
NLR	0.06	0.004	NSV	0.06	0.003
TKC	0.06	0.013	RRC	0.06	0.008
MDY	0.06	0.006	DTQ	0.06	0.004
CDC	0.06	0.035	NFK	0.06	0.004
MNG	0.06	0.005	NFR	0.05	0.004
NNE	0.05	0.004	NKK	0.05	0.004
DVS	0.05	0.003	DDS	0.05	0.003
DAT	0.05	0.003	MDI	0.05	0.005
NRV	0.05	0.003	PVD	0.05	0.003
DHR	0.05	0.004	NSQ	0.04	0.004
PFE	0.04	0.003	DDQ	0.04	0.004
DLH	0.04	0.004	DQT	0.04	0.004
NLK	0.04	0.003	NNH	0.04	0.006
PNF	0.04	0.003	DAS	0.04	0.003
NSM	0.04	0.006	KNK	0.04	0.002
NKV	0.04	0.003	NRR	0.04	0.004
DIK	0.04	0.003	NFH	0.04	0.006
NDN	0.04	0.004	DTK	0.04	0.003
DVT	0.04	0.003	DNW	0.04	0.004
MSF	0.04	0.006	HFM	0.04	0.018
MDE	0.04	0.006	CSC	0.04	0.036
EDK	0.04	0.003	NYH	0.04	0.006
DQK	0.04	0.004	PTD	0.03	0.004
DFR	0.03	0.004	DVK	0.03	0.003
MAG	0.03	0.005	PYE	0.03	0.003
PND	0.03	0.004	DSH	0.03	0.005
HAM	0.03	0.011	MQG	0.03	0.004
DKT	0.03	0.003	PNV	0.03	0.003
DAH	0.03	0.005	SNK	0.03	0.002
MRG	0.03	0.005	PNA	0.03	0.003

Triads	Relative Entropy w.r.t X_1GX_3	Confidence Interval	Triads	Relative Entropy w.r.t X_1GX_3	Confidence Interval
MNI	0.02	0.005	DHK	0.02	0.004
PTC	0.02	0.016	PDD	0.02	0.004
DCS	0.02	0.004	NYR	0.02	0.005
DTC	0.02	0.007	NTR	0.02	0.004
DVR	0.02	0.003	TNK	0.02	0.003
DWK	0.02	0.004	ENV	0.02	0.002
DLS	0.02	0.003	CDP	0.02	0.008
MDF	0.02	0.006	QNV	0.02	0.003
SHM	0.02	0.005	DHS	0.02	0.004
LDI	0.02	0.002	NLQ	0.02	0.004
GNM	0.01	0.004	CHT	0.01	0.007
HYY	0.01	0.006	PID	0.01	0.004
PYD	0.01	0.004	NKQ	0.01	0.004
NAH	0.01	0.006	PDT	0.01	0.003
DRR	0.01	0.004	KNE	0.01	0.003
NRH	0.01	0.006	DRK	0.01	0.004
CSR	0.01	0.007	DTS	0.01	0.003
ENK	0.01	0.003	NRK	0.01	0.004
MLG	0.01	0.004	DVC	0.01	0.005
NIC	0.01	0.007	HNY	0.01	0.006
YSM	0.01	0.006	NDQ	0.01	0.004
DYN	0.01	0.003	NNT	0.01	0.003
MDK	0.01	0.005			

Supplementary Table S3. Relative entropy (=($S(X_1X_2X_3) - S(X_1GX_3))/S(X_1GX_3)$) of triads ($X_1X_2X_3$) which are least entropic (top 100 triads) w.r.t X_1GX_3.

Triads	Relative Entropy w.r.t X_1GX_3	Confidence Interval	Triads	Relative Entropy w.r.t X_1GX_3	Confidence Interval
HEC	-0.93	0.015	WCL	-0.93	0.010
IWM	-0.93	0.009	HMC	-0.93	0.015
MEC	-0.93	0.015	NMC	-0.93	0.012
QCD	-0.93	0.008	KMW	-0.93	0.009
CPW	-0.93	0.014	MRW	-0.94	0.012
WYC	-0.94	0.051	QCY	-0.94	0.008
WNW	-0.94	0.012	NCC	-0.94	0.012
CMP	-0.94	0.012	WCF	-0.94	0.011
CYP	-0.94	0.011	FCW	-0.94	0.011
KMC	-0.94	0.010	MCQ	-0.94	0.011
WMI	-0.95	0.010	WFC	-0.95	0.036
CWK	-0.95	0.009	FCC	-0.95	0.010
MWI	-0.95	0.010	NWM	-0.95	0.010
CCK	-0.95	0.010	MWR	-0.95	0.010
MWG	-0.95	0.009	SCM	-0.95	0.010
KCM	-0.95	0.007	WCQ	-0.95	0.009
NCW	-0.95	0.009	ICW	-0.95	0.009
CWE	-0.96	0.008	CMK	-0.96	0.008
WWP	-0.96	0.009	SCN	-0.96	0.008
EMW	-0.96	0.008	FCM	-0.96	0.008
MCT	-0.96	0.008	MHM	-0.96	0.008
LMC	-0.96	0.008	TCW	-0.96	0.008
SMC	-0.96	0.008	CDH	-0.96	0.008
CMG	-0.96	0.008	GMC	-0.96	0.007
WEC	-0.96	0.033	WTC	-0.97	0.036
WCY	-0.97	0.006	RMW	-0.97	0.006
MCF	-0.97	0.006	MQC	-0.97	0.011
PWC	-0.97	0.009	WHW	-0.97	0.009
WLC	-0.97	0.029	CTM	-0.97	0.010
FMM	-0.97	0.005	HPC	-0.97	0.009
WSC	-0.97	0.030	CDW	-0.97	0.008
MWE	-0.98	0.005	FMC	-0.98	0.007
WAW	-0.98	0.007	CPM	-0.98	0.008
MCY	-0.98	0.007	HWM	-0.98	0.008
HKC	-0.98	0.008	MIM	-0.98	0.006
MPW	-0.98	0.006	CEW	-0.98	0.007

Triads	Relative Entropy w.r.t X_1GX_3	Confidence Interval	Triads	Relative Entropy w.r.t X_1GX_3	Confidence Interval
CMQ	-0.98	0.006	WCH	-0.98	0.006
CCQ	-0.98	0.006	CTC	-0.98	0.012
MCV	-0.98	0.005	PWW	-0.98	0.005
WWA	-0.98	0.005	CMY	-0.99	0.005
QMW	-0.99	0.005	CWG	-0.99	0.005
CWT	-0.99	0.004	MWP	-0.99	0.005
WCE	-0.99	0.004	SWC	-0.99	0.005
WMY	-0.99	0.004	YYC	-0.99	0.004
MWA	-0.99	0.004	CCM	-0.99	0.006
PCH	-0.99	0.003	YCH	-0.99	0.003
CHM	-0.99	0.005	TCC	-0.99	0.004
WPW	-0.99	0.004	CYW	-0.99	0.004
MPM	-0.99	0.003	CKC	-0.99	0.007
KCC	-0.99	0.003	CMM	-0.99	0.004

PDB IDs of 1917 Globular Proteins:
2DY7A, 4A5VA, 1BR0A, 2H2MA, 1GH9A, 2L52A, 3HVVA, 1JO6A, 2RQ5A, 2BZTA, 2KVLA, 2DGYA, 3C12A, 1DHNA, 2LS01, 2KMVA, 2LAVA, 1HE9A, 1ICXA, 2IWCA, 1JLNA, 3FS5A, 2EYIA, 2KXFA, 2W0IA, 2KNAA, 2OJ4A, 2CQWA, 2EZLA, 2KYWA, 2M2DA, 2JSPA, 3MZZA, 1GTAA, 1WOTA, 1IRLA, 2KAFA, 2LSEA, 2D1UA, 2PP4A, 1BYWA, 2HWTA, 1JW3A, 2W0NA, 2B0AA, 2JMLA, 1H7DA, 1M9GA, 2SILA, 1ABZA, 2KO6A, 2K0SA, 2JA4A, 1BKBA, 2E6JA, 1ZITA, 2YVNA, 1D6BA, 1K42A, 1L3YA, 1BW3A, 2LC2A, 1WFOA, 2K07A, 2DI0A, 2BO5A, 2IF1A, 2DZLA, 2DN8A, 1QJ9A, 2L9QA, 1ZHVA, 1YDUA, 1TH5A, 2DGZA, 2ROVA, 2LNAA, 1NGLA, 1IFGA, 2EDUA, 2NS5A, 1WMTA, 1Z33A, 1TZQA, 1UB9A, 2FYJA, 1IRZA, 2L9MA, 1A8OA, 1ZLBA, 2LRUA, 2JY9A, 2LTUA, 1SS6A, 1EXGA, 1J7GA, 1WHQA, 1L1PA, 1WXPA, 2KY9A, 1B64A, 2E00A, 1RLFA, 2JMSA, 1VDLA, 1K40A, 2E6ZA, 1TM9A, 2L7SA, 6XIAA, 1VD0A, 1T0GA, 2L0RA, 4BHCA, 1EDGA, 3APPA, 2EKHA, 1CX1A, 1YQ8A, 2VXDA, 2K0NA, 2A55A, 1IFPA, 1TAPA, 2KLUA, 1YT4A, 1GP8A, 1UGLA, 1BNOA, 2KRXA, 1R3BA, 2KMLA, 1X1FA, 3B02A, 1G8AA, 2EAOA, 2HTJA, 1P1DA, 2C0WA, 1AHCA, 2J4MA, 2KVPA, 1XRDA, 2DAFA, 1OVQA, 2JQXA, 1CDBA, 2YWXA, 1YZSA, 2JR0A, 1OLRA, 1UQVA, 2KCWA, 1LPLA, 2CRLA, 2LU3A, 1ALDA, 2LONA, 1QJTA, 2M3KA, 1U3NA, 2LBOA, 1DUJA, 2LC3A, 4DOMA, 2OYZA, 1BIPA, 2KA0A, 2L8OA, 2KRKA, 2KW7A, 2CQUA, 1EQ1A, 2FQHA, 3IHOA, 2KIAA, 1BRZA, 3V5BA, 2LEMA, 2D9ZA, 1JWEA, 2KXSA, 1KVPA, 1J57A, 2D8MA, 2DW3A, 2LXFA, 1CEMA, 2YSEA, 2KNJA, 1GJSA, 3FH2A, 3MXXA, 2DBCA, 1KUUA, 1TGOA, 1WH0A, 2AH5A, 2LQKA, 1Y2YA, 2V0FA, 1TQZA, 1QR9A, 2RA1A, 1FTSA, 3D4MA, 4E5RA, 1V49A, 1JXCA, 2LTLA, 2LNTA, 1Q50A, 2KONA, 2K3PA, 1HKGA, 1DTZA, 2KXYA, 1DVOA, 2WCYA, 2LJPA, 2L4WA, 1DDFA, 2V14A, 1WJ4A, 2BIDA, 2EDMA, 1WFMA, 1POUA, 1H8MA, 2KUMA, 2K0MA, 1Y7XA, 2PETA, 1YWXA, 2KSWA, 1DROA, 1QQFA, 3AAPA, 1L2PA, 2KMGA, 1UAPA, 2M5OA, 1OJQA, 2JXTA, 3LD1A, 1BD8A, 2JO1A, 2KMFA, 1N5HA, 2KCKA, 1SXMA, 1X58A, 1Q48A, 1C2AA, 1FQQA, 2YU3A, 2E5EA, 1G6EA, 1USSA, 3JV1A, 1VFIA, 2K88A, 2K2EA, 1NEQA, 1ZTSA, 2B68A, 2LCUA, 2OF3A, 2KBZA, 1F7WA, 1H09A, 2L0KA, 1T4YA, 2CTXA, 2XV9A, 1FUSA, 1X3AA, 2CR9A, 1C3GA, 2K3AA, 2M09A, 1Q1OA, 2EQNA, 1K8HA, 2YUIA, 1PLRA, 1EYHA, 3VPYA, 1ZRXA, 1BU2A, 2M4FA, 2LXNA, 2JNSA, 1C3YA, 2RRUA, 1ZLGA, 2ADZA, 4DNUA, 1EZAA, 1X4PA, 2XXSA, 1SG5A, 1WS6A, 3PG4A, 2LFPA, 1BEOA, 1DQCA, 2KMZA, 1P4SA, 2Z9TA, 2JVJA, 1D2PA, 4B50A, 1P3CA, 2KZ4A, 1HA8A, 4FCUA, 1W9RA, 1NYOA, 1KV4A, 2L82A, 3E9UA, 2E3EA, 2K75A, 2KGSA, 1OH1A, 2YSZA, 1WJ5A, 1PBUA, 1PMDA, 2JOQA, 2EDOA, 1WISA, 2KPTA, 2L06A, 2GQCA, 1POQA, 2AYYA, 2KMUA, 2KCOA, 1R5SA, 2JTCA, 1H6TA, 2KHVA, 1V5MA, 2LG1A, 1AD6A, 2BNHA, 3IV4A, 2AC4A, 2YUYA, 1J8SA, 1DQ0A, 2VU5A, 1ZLMA, 2R0SA, 2KWHA,

1D8BA, 1MM4A, 1BSYA, 2KRUA, 2JR3A, 1EO1A, 2J5OA, 4GLPA, 3FK5A, 2HO9A, 2LNLA, 2I85A, 3RZYA, 2CRQA, 2LLZA, 1VGJA, 1WLMA, 2L60A, 2CR4A, 3N4UA, 1NI5A, 2A2PA, 2ZGKA, 2PTHA, 2DYIA, 1H5OA, 2L76A, 2L2QA, 1HS7A, 1NG6A, 1V5TA, 3JZZA, 2CQJA, 2RPBA, 1DE3A, 2G31A, 4K14A, 2KUBA, 1XSAA, 1UFMA, 1JEJA, 2KHKA, 2IKEA, 1TTYA, 1RTCA, 1UCSA, 2CUJA, 1XDWA, 2KZXA, 1P6RA, 2L8YA, 2K8QA, 1ZEQX, 1XUTA, 2CSPA, 1CIXA, 2QHTA, 2LPEA, 2KZ9A, 2K3GA, 1ZK9A, 2EE0A, 2LCDA, 2GE4A, 2FFTA, 2LF2A, 1UTGA, 2KJ6A, 2JUBA, 2NEFA, 1WGRA, 2KNGA, 2JO7A, 2KSYA, 1MUFA, 1P9CA, 2JT3A, 2KK9A, 2JTMA, 2LWFA, 2AI6A, 2C08A, 3IPZA, 2LYIA, 1ERCA, 1CWXA, 2K3NA, 2KTAA, 2DO9A, 2VIKA, 2JR1A, 1VSMA, 1EDQA, 2AHNA, 1DABA, 2DB1A, 1WHBA, 2K1SA, 2KVVA, 2PFUA, 2DKSA, 2JWPA, 1EHDA, 2LFIA, 1GXIE, 1CRZA, 1PFJA, 2WGNB, 2W9OA, 2DLLA, 2GFUA, 1A91A, 1UEOA, 2KQUA, 2M4MA, 3EZMA, 1VPUA, 2LOEA, 2YZTA, 2RRFA, 2BAFA, 2DMWA, 1RCBA, 2LQXA, 3UAFA, 3QPAA, 2BVVA, 1V60A, 1E6MA, 2EJEA, 2LSMA, 1G2HA, 2RRNA, 2E34A, 3CH7A, 2W9QA, 2RQRA, 2FRGP, 2L4AA, 2KE4A, 2H3KA, 2K9SA, 3CRDA, 1B8WA, 1SS1A, 2LBCA, 2H0MA, 4GIFA, 1YWYA, 2LUUA, 1WVKA, 4FEIA, 2JRYA, 2LRAA, 1WH9A, 3FHFA, 2J8PA, 1Z4HA, 2YRUA, 2OA4A, 2QEVA, 2LE3A, 1X0MA, 2JUOA, 1UGOA, 2GGOA, 1AG4A, 2GO9A, 2IEMA, 2LVLA, 1HOEA, 1Z2QA, 2KD2A, 2KFWA, 2KBQA, 1D4BA, 1GWPA, 2OV6A, 1AB3A, 2KMWA, 2M0AA, 2IGDA, 2DO5A, 2KISA, 1PQNA, 1JAJA, 2KSGA, 2LMKA, 2CQLA, 1SRVA, 1KS6A, 2HX6A, 1Q0VA, 2CQ7A, 1WPIA, 2JX5A, 2LNBA, 2JYNA, 3BWTA, 1BV8A, 1M12A, 2LZ0A, 2KUTA, 1SSNA, 2M4LA, 2KL7A, 3ACPA, 1ESRA, 1RZSA, 2K32A, 2LFKA, 2LKYA, 2P0PA, 2KBBA, 2IVWA, 2TGIA, 1YHPA, 1WT7A, 2FUIA, 1JDQA, 2KTLA, 1T1DA, 1L2MA, 2LGTA, 2P52A, 1HX2A, 1WWQA, 2M5ZA, 2A3DA, 1BT9A, 1Z1MA, 1TPMA, 2HC5A, 1VLSA, 1O8RA, 2L6OA, 1CEWI, 2E62A, 1WH5A, 2RQ8A, 1ADXA, 2LAIA, 1R6HA, 1UX5A, 1L8FA, 3KVDD, 2EH0A, 2LJ2A, 1LWRA, 1H5PA, 1W2QA, 2F82A, 2LLGA, 2ROOA, 2DK3A, 1Z9IA, 1WF6A, 2E2FA, 2Q98A, 2IFOA, 2KL8A, 2CSOA, 2RMJA, 2K8YA, 1MO7A, 1K0FA, 2LIYA, 2Y0RX, 2O0I1, 1DGUA, 1FPSA, 1QAUA, 2JM2A, 1HYKA, 2JSOA, 2D35A, 2K4VA, 1ILOA, 1GPSA, 1BEDA, 4PTIA, 2Q6ZA, 3ZUAA, 2LAHA, 3H6JA, 1RESA, 1JT8A, 2LM0A, 2PLCA, 2ENJA, 2KN1A, 1PCPA, 1BZ4A, 1UG2A, 1ZGGA, 1F4IA, 2P6JA, 2DOFA, 1F6VA, 1GA3A, 2L0CA, 2CW4A, 1KKGA, 2B8IA, 1YZFA, 1TBDA, 2L8AA, 2I6VA, 1KOYA, 1WKTA, 2I3EA, 1CIIA, 1HICA, 2KXIA, 1L5CA, 2KT7A, 2PNDA, 1CZ1A, 1EQ7A, 1RKMA, 2YZ0A, 2K37A, 2LQJA, 2M3DA, 1KLOA, 1WJ6A, 1DIXA, 1ND7A, 1Q1VA, 2GYTA, 1P9KA, 1UGJA, 1YUAA, 2GDTA, 2KCDA, 1K0SA, 3KTTB, 2DJVA, 2LA3A, 1H9EA, 1KF5A, 2COBA, 2JRKA, 2KS6A, 2EKFA, 1SOUA, 1C8PA, 1P7SA, 2GAQA, 1X53A, 2JZ2A, 1UG1A, 2KMCA, 1JHSA, 2LHRA, 1DVCA, 2LC0A, 1UH6A, 1BM8A, 3V1AA, 2L1NA, 2RR7A, 2KBFA, 2JONA, 2KD7A, 1X3QA, 1WLXA, 1WLOA,

1DUSA, 2KRTA, 1B1AA, 2AIVA, 4F91B, 1JJGA, 3M70A, 1IQOA, 3RDJA, 2X8XX, 2GO0A, 1OF9A, 1S79A, 1WFIA, 2L91A, 2LT2A, 1UTAA, 2K4MA, 2EEFA, 1OOIX, 2L8DA, 2LKLA, 1P68A, 1ZO0A, 1N7OA, 2KQ5A, 2E7GA, 2L7JA, 2HGKA, 2JQAA, 2DNXA, 2GKTI, 2KF2A, 2KVOA, 2D3JA, 1CQYA, 1POZA, 1CBGA, 2DK4A, 1ITPA, 1QTFA, 1D8JA, 2PIIA, 2FD5A, 2HHIA, 1UJLA, 2FCDA, 1C05A, 2JV0A, 1R73A, 2YUFA, 2JXUA, 1KG1A, 2EBIA, 1HK6A, 2X9ZA, 2CTKA, 1OCPA, 2L8BA, 2L03A, 3RJPA, 1K1VA, 2JOEA, 2HDEA, 2KSLA, 2JNZA, 2LNJA, 1U97A, 1I35A, 3CBHA, 2KI8A, 2M1ZA, 2JSWA, 2M7OA, 1TUZA, 1QU5A, 2L6AA, 2CPRA, 1SCZA, 1XSFA, 2Y9TA, 3TOWA, 1O8MA, 1XQ8A, 2KDOA, 1WWYA, 2EE7A, 1DIVA, 1SZLA, 1J2MA, 2L37A, 2L2DA, 2ORCA, 1QU6A, 2DO3A, 2LT8A, 3G4PA, 2L7KA, 1Q2YA, 2NSCA, 2KJFA, 1PSYA, 2DIWA, 2GUTA, 1V05A, 1TOZA, 2LENA, 1CM3A, 1QL1A, 2B4NA, 1JJRA, 3GV3A, 1WJNA, 2LGNA, 2H7AA, 2JN6A, 1N4CA, 1XGDA, 1HN6A, 2LM4A, 3S0AA, 2KYZA, 2WGOA, 1JVRA, 2KWZA, 2LNVA, 2BK8A, 2DHXA, 1EIWA, 1A5JA, 1I5PA, 2KN8A, 1Y5OA, 2LSYA, 2DP5A, 2A6ZA, 3ND2A, 2KPQA, 1F2HA, 1GBSA, 2LUQA, 2E8FA, 2L3LA, 1WFQA, 2CQYA, 2CQ8A, 2JS4A, 2LXRA, 2AZPA, 2KY6A, 1P7NA, 1G84A, 2HF6A, 1WI9A, 1WJJA, 2BZMA, 2LCLA, 1BB8A, 2K36A, 2YXPX, 1IV8A, 1Z8MA, 2JQVA, 2KCZA, 1SSLA, 2J5HA, 1X3UA, 1HY9A, 2K54A, 3ENUA, 2JPUA, 1FSFA, 2DAEA, 2C6UA, 1EMRA, 2GLWA, 1CMOA, 1X9BA, 2L9FA, 2LVGA, 1RQ6A, 2DY8A, 2COKA, 1C93A, 1ILYA, 2ENKA, 2KREA, 1YBJA, 2DP9A, 1BETA, 1IJBA, 2VXEA, 1UG4A, 1JI8A, 2KZRA, 1BHUA, 1RMJA, 1NR3A, 2H2ZA, 1USGA, 3RFFA, 2CYGA, 2YUSA, 2OUTA, 2DBAA, 1V95A, 2P3MA, 2AFDA, 1WFRA, 1HN3A, 2K86A, 2DALA, 2GQBA, 2DNYA, 1CDZA, 2P30A, 1BY1A, 1ARLA, 1ILKA, 2L1IA, 1SR2A, 2KR6A, 2KGLA, 1WI0A, 1WJWA, 2JQGR, 2JSXA, 1AAJA, 1VEEA, 2RNGA, 3OZHA, 2K87A, 2JTVA, 2DAJA, 2KIGA, 2DO7A, 2LQUA, 1QQHA, 1DP3A, 1FSBA, 1MYNA, 2LVSA, 1QA5A, 1JRMA, 1WJ7A, 1QCXA, 1JBIA, 1F94A, 1H2PP, 2LQ7A, 3TUAA, 2AGAA, 2KL5A, 1XS8A, 4A4DA, 1T23A, 2KC6A, 2KK0A, 3CSRA, 1APQA, 1F00I, 1P1LA, 1WP5A, 2KKJA, 2KBIA, 3BN6A, 2OV1A, 2LDKA, 1JLIA, 2KCAA, 1GYDB, 1KVNA, 2EE2A, 2YRBA, 1Z2FA, 2CQMA, 2K89A, 2LAAA, 2P01A, 1UCUA, 2RN7A, 2BW2A, 1V65A, 1HH2P, 2LN8A, 1O8TA, 1VD2A, 1K89A, 1QO6A, 1R6EA, 2K9MA, 1PXUA, 2L3BA, 3PTJA, 2CH0A, 1AB7A, 1PQXA, 1AHLA, 1TNSA, 1N87A, 2DB2A, 2JX4A, 3KT9A, 3ILSA, 3TIPA, 1TQGA, 1H9FA, 1WK0A, 2LG4A, 1GHHA, 1U89A, 2K9PA, 1KSQA, 2HLQA, 1ENHA, 1DZ7A, 2K6HA, 2H8EA, 1Y2QA, 2JW5A, 1H6QA, 2L5CA, 3ZG4A, 2LOTA, 2K4QA, 2IE8A, 2O4EA, 2JXPA, 1EJGA, 2KFPA, 1ZZAA, 2JUJA, 1EW3A, 1V32A, 1BYIA, 3LJ8A, 2KZWA, 2LH9A, 2KESA, 2KJVA, 1L1IA, 1FAFA, 2GT5A, 2EO2A, 1NKLA, 1KJUA, 2FBOJ, 2KJGA, 2L6QA, 1F53A, 1GJXA, 2K5HA, 1Q80A, 2L0DA, 2KW5A, 1S29A, 2KZ8A, 2L3NA, 2K2OA, 2KL4A, 2KJYA, 2NWDX, 2ITHA, 1J0SA, 2LW6A, 1GH8A, 1WYJA, 2LFVA,

2LORA, 2DMEA, 2JZFA, 1G03A, 2KZSA, 2G0UA, 2BAAA, 2KSVA, 2A4HA, 1CDHA, 1YWLA, 2KSFA, 2RM5A, 2M4GA, 3F9VA, 2LPDA, 1WINA, 1EMWA, 2COOA, 2RSOA, 2F3IA, 2LYCA, 1SLLA, 2YUGA, 2DA6A, 2K18A, 2LLMA, 2LF6A, 2KPYA, 1GLNA, 1I2UA, 2JN9A, 1FYBA, 2JSFA, 1ANUA, 1XIXA, 1PNEA, 2FMRA, 2FCBA, 1K5KA, 2CU1A, 2GPQA, 2JQ3A, 4AKAA, 2JZ4A, 1CFIA, 2LEQA, 2LMZA, 2KOUA, 2KB9A, 2LIOA, 1PWKA, 1XEEA, 1X67A, 1N7LA, 2LLNA, 2L1SA, 3KJEA, 1GO9A, 2KKXA, 2BYEA, 1DT4A, 1LL8A, 2RSGA, 2V6CA, 2YQKA, 2L3UA, 2CJNA, 3A7LA, 3UCIA, 1YXEA, 2EZIA, 2KVSA, 1ZXFA, 2JTOA, 1SURA, 2L25A, 2LPIA, 2KPMA, 2K5GA, 1WRGA, 2K3DA, 2K8OA, 1CWYA, 2KUAA, 1G7EA, 1WCLA, 1X1EA, 1I6ZA, 1MN3A, 2AGMA, 1FEXA, 2KEPA, 1MD6A, 1RW2A, 2M26A, 2LLVA, 2ROQA, 1DV5A, 2HEQA, 2K2BA, 1SKFA, 1D3LA, 2FJ6A, 1NE3A, 1TVMA, 2BICA, 2LGZA, 3SH4A, 2D56A, 2KYYA, 1PMRA, 2KWAA, 1AK6A, 2CWYA, 2E0GA, 2PXGA, 1R4GA, 1GGVA, 1FASA, 1JCUA, 1P6TA, 2L7WA, 2CRFA, 1GOKA, 3RA2A, 2KK7A, 2LR4A, 2L8KA, 1HNRA, 1ZC1A, 1OGWA, 2IMUA, 2AXLA, 2KCNA, 2KYHA, 1QZMA, 1G7OA, 2K5IA, 2KVTA, 2KHEA, 1LWBA, 1XPNA, 2KLBA, 1N81A, 2DKZA, 2LRGA, 2L2EA, 1ND9A, 1WG7A, 1WH8A, 1HP8A, 4A02A, 2JWYA, 2GMGA, 1BUYA, 2K4KA, 2K3IA, 1WHNA, 1WQKA, 2KPPA, 1YR1A, 2HNAA, 1HDLA, 2YS4A, 2QU4A, 2L57A, 1PVEA, 1HKYA, 1DTVA, 1IO2A, 1IB8A, 2KJRA, 2KW8A, 2RQYA, 1AUZA, 2JHBA, 1V2YA, 2KZ5A, 2JPNA, 1I1SA, 2SNVA, 1MF7A, 2KE3A, 1KMXA, 2OQPA, 1EIKA, 2KQPA, 1AHOA, 1MHNA, 1HGVA, 2YS0A, 1VIBA, 3PM2A, 2L04A, 2BKDN, 1A6CA, 2FC3A, 1IS1A, 1A8QA, 1OMPA, 1PM6A, 3HAKA, 2JTYA, 1RHDA, 2JQYA, 1NXIA, 1WK1A, 1T45A, 2LQ4p, 2FJLA, 1C44A, 1YPCI, 4HJCA, 2L1PA, 6RHNA, 1LNSA, 2LCYA, 1RQSA, 1X4TA, 2D46A, 2IUEA, 1QXXA, 1SAPA, 1G62A, 2E63A, 1VDIA, 1QTTA, 2CR7A, 1GM0A, 1T8CA, 3HSFA, 1XU6A, 1UFZA, 2K49A, 2JWGA, 1JBJA, 2RH3A, 1TDPA, 1WWIA, 2KPUA, 1SORA, 1AGXA, 1SBXA, 1KJ6A, 1L6HA, 2E8PA, 2BBXA, 1H4AX, 2GPRA, 1IVZA, 2PTLA, 4DNIA, 2IN2A, 2LMDA, 2LRSA, 1X0HA, 2K7MA, 1VCCA, 2C83A, 1N3HA, 2EE5A, 2KLQA, 3O04A, 2JOVA, 2BFHA, 1VGPA, 1V61A, 2L9DA, 1X3OA, 2F76X, 1HCDA, 2NOCA, 1MR4A, 2E70A, 1M42A, 4ACJA, 2EQOA, 2BVBA, 1AJEA, 2KVAA, 1GYFA, 2NRGA, 1NOAA, 4A53A, 2LL0A, 3DJ9A, 1SSKA, 1RC9A, 2K5PA, 2EKIA, 1IW4A, 1X5BA, 1QW1A, 1QW2A, 2K6GA, 2B3WA, 2KK2A, 2SGAA, 1AKZA, 2JMBA, 1MIXA, 2D5UA, 1IIOA, 2KQ6A, 8OHMA, 1CTOA, 2DJPA, 2E7MA, 2QMVA, 2LYXA, 1YYCA, 2GLEA, 1X0OA, 2KYTA, 2K5EA, 2EVNA, 2LRDA, 2LW3A, 2FJZA, 2K1AA, 1PBNA, 3T7FA, 2DJ1A, 2KT8A, 3JZ9A, 1T2IA, 1WJUA, 1LMZA, 1WITA, 2LQ8A, 2AYAA, 1K19A, 2L08A, 1PRZA, 1Z8SA, 2R6QA, 2K9DA, 2NSNA, 2ARAA, 2VQ4A, 1LKNA, 1G7DA, 1HREA, 1H3QA, 2DI7A, 2KOKA, 3A4CA, 2DAQA, 2JHYA, 2G69A, 2ESKA, 1PC0A, 1J7XA, 1OTPA, 1XAAA, 2KA5A, 2RNNA, 2FZ0A, 1K8VA, 1GYVA, 1BSHA, 1AKOA, 2PNEA, 2QZQA, 2KK4A, 1WYMA,

1ACFA, 1FNAA, 1X51A, 2DCVA, 2L6NA, 1U09A, 1BUOA, 2KMAA, 2COMA, 1WJTA, 1SS3A, 2LA7A, 3HVMA, 1WHIA, 2L3TA, 1ZWTA, 1N3GA, 1WGOA, 2PKOA, 1BBGA, 1TKNA, 1NE5A, 2C7HA, 2KX2A, 1LXAA, 2KQRA, 1PS2A, 1UJRA, 1EHSA, 1YU5X, 2DAWA, 1IUFA, 1PGYA, 1J0TA, 2GGFA, 2DO8A, 2P25A, 1W0BA, 2KD1A, 1IMTA, 2YRQA, 1ALYA, 1FHOA, 2K73A, 2UWQA, 2RSXA, 3UFCX, 2JOZA, 2JZAA, 2M4YA, 2CRUA, 1MJZA, 2HJQA, 2JNKA, 2JOBA, 2JPSA, 1O5TA, 2L5QA, 2YWOA, 1BI5A, 1IEZA, 1URFA, 1T0VA, 1QKLA, 3FI1A, 1YVCA, 3ETPA, 2V1NA, 2LJKA, 1V5SA, 3JYLA, 3CO1A, 2RR8A, 1QTSA, 2LQGA, 2DIUA, 2K8PA, 2JVGA, 2DIRA, 1KIVA, 2K4ZA, 1QK9A, 2BZEA, 1AIWA, 2HVMA, 1BDSA, 4E2UA, 2KZ0A, 1RHXA, 1T17A, 1YSYA, 2C55A, 2KCQA, 2KZ3A, 1GQZA, 1OWAA, 1YUBA, 1EGXA, 1FHLA, 2KJQA, 4J5QA, 2YV0X, 1LQCA, 2J9VA, 1A63A, 1Q59A, 2CR2A, 1HHNA, 1K8BA, 1GVPA, 2K24A, 2YGSA, 3DJNB, 2RSTA, 1FGPA, 2K5SA, 2KRCA, 1ZDVA, 1EHXA, 2HE7A, 2CP8A, 1APCA, 2KHQA, 3R6DA, 2EQXA, 2YRVA, 2KEQA, 4AQZA, 2PPNA, 1MSCA, 1WH2A, 1F5XA, 1GVLA, 1QTOA, 1UDMA, 2LSHA, 2HZDA, 2DIQA, 1UOYA, 1WM3A, 2LVXA, 2KT6A, 2ICTA, 3DPAA, 1IADA, 1UKFA, 1WGBA, 2RRLA, 1M2EA, 1B75A, 2CPTA, 3M66A, 2L81A, 1L7YA, 3E7RL, 2I8LA, 2KC5A, 1RYKA, 1GHTA, 2LV4A, 2AKKA, 3PFMA, 3LFOA, 2DKQA, 2LNMA, 2DUNA, 1EOVA, 1CKRA, 1UFGA, 2KJIA, 1LQ7A, 1G5ZA, 2LQ3A, 2FXTA, 2X43S, 1BVHA, 1XKEA, 1WHRA, 2JZ6A, 1SE7A, 4J4RA, 2V31A, 2CG7A, 1GYZA, 1NFAA, 2JSNA, 2JZTA, 1ZAQA, 2LN7A, 1G9PA, 1BHEA, 1KOEA, 2KQZA, 2DLXA, 2M6YA, 1UG7A, 1Q38A, 1TJDA, 2RKQA, 2GF5A, 2VKCA, 2K5QA, 3IH4A, 2XK0A, 2CASA, 1MZLA, 1EPSA, 2DBHA, 2CQAA, 2LHTA, 2PY1A, 1KJSA, 2DBGA, 2J6BA, 1FYXA, 3NPOA, 2RM8A, 1XN7A, 1OXDA, 1DOVA, 1IOJA, 1EWIA, 1PBVA, 2RN4A, 1RL0A, 1AJMA, 2FPHX, 1QSVA, 1UF0A, 2IFSA, 2LFCA, 1TE7A, 2VIMA, 2B5XA, 1ON4A, 1EW4A, 2KMSA, 2KATA, 2M4HA, 1N6UA, 2LNCA, 2L1QA, 2L2CA, 2JVMA, 1HKAA, 2LKGA, 1E8RA, 1N6ZA, 2B9KA, 1V9VA, 2BK2A, 1C01A, 2KKVA, 2ENQA, 2QT4A, 3MX7A, 2KNIA, 2KRHA, 1WXMA, 1I2TA, 1QM9A, 1ATAA, 2KJXA, 1WRYA, 1K0HA, 2FB9A, 1N91A, 2FMCA, 3N0KA, 2DHZA, 2D49A, 2OKTA, 2KWYA, 1PV0A, 4AOGA, 1BQCA, 1GO5A, 1Z9BA, 1ZHCA, 3ZPMA, 2DHSA, 1NHLA, 2WQGA, 2KUQA, 1E0HA, 1N88A, 1I60A, 1MWPA, 4ETXA, 3ZSLA, 2LY8A, 1WJKA, 3QYUA, 2M4VA, 1BZKA, 1T50A, 2K4NA, 1WJRA, 2CKXA, 1CKVA, 1EJ5A, 1ZUGA, 2KL2A, 1ERDA, 2KGFA, 1TJEA, 1LXLA, 2M47A, 1JI6A, 1R36A, 2JOYA, 2KEYA, 1WXAA, 2DOAA, 4JZCA, 1X52A, 1WKIA, 1XWEA, 2L1AA, 2UWRA, 1CEJA, 2L1TA, 1XNEA, 1P4XA, 2JXWA, 2Z4DA, 1A9VA, 1F6WA, 1WICA, 1YO4A, 4ULLA, 1O6XA, 3KB5A, 1KS9A, 1CZ4A, 2GBSA, 2JWHA, 1DVNA, 2K0RA, 2K5RA, 2YUDA, 1UHUA, 1JYTA, 1UG8A, 1V63A, 2KK1A, 2KLLA, 2DAVA, 1YALA, 1BGFA, 2RRDA, 2LOJA, 3SEBA, 2KNQA, 4AR0A, 2LN3A, 1ESXA, 1RZWA, 1MV3A, 2KIWA, 2JYEA, 2DA7A, 2KQ1A, 3MTVA, 1AYDA, 3T5BA, 1AGGA,

2H85A, 2K0QA, 1WF9A, 1EDXA, 3H8NA, 1FMYA, 1WGPA, 1COUA, 2YTUA, 1QHKA, 2C0SA, 1RIPA, 2CRVA, 2K4FA, 2K27A, 1Q2UA, 1A6SA, 1OQAA, 1S7IA, 2RM4A, 1RY3A, 2LAOA, 3VORA, 1YNXA, 2Q34A, 1MJCA, 1HJ0A, 1IP9A, 3F7MA, 1N3KA, 1WIXA, 2LAEA, 1A3HA, 2RNOA, 1WU3I, 3DQPA, 1WGWA, 2LWYA, 3H7ZA, 3LIGA, 1PC2A, 1R8NA, 1SB6A, 2E60A, 1WH4A, 1MP1A, 2K4EA, 2L95A, 2TMPA, 1YUWA, 3NSMA, 1VZSA, 1WWBX, 2K19A, 2COSA, 2RLOA, 2JV8A, 1YW5A, 2M0XA, 1ZU2A, 1U5MA, 2K13X, 1Q5FA, 2W0GA, 1OJGA, 2FE9A, 1AOYA, 3ONJA, 2LTFA, 2LD3A, 2ERFA, 2KQ2A, 2CTQA, 1BBYA, 1WWUA, 1LMIA, 1AA3A, 2LUWA, 1Y6IA, 2PLFA, 1PDBA, 2JMUA, 1WGSA, 2LG7A, 2OT2A, 1PJBA, 2OHEA, 2LIEA, 2KT9A, 2JA9A

PDB IDs of 138 Group I Proteins:
2ZKQq, 4KZYe, 2H4MC, 3IOZB, 4IJ3C, 3IFNP, 3ZMSC, 3BBO6, 4KP3E, 3TPMB, 3DBOA, 3KL4B, 3GZ2P, 4C2M4, 2JDII, 4GMNB, 2M14B, 2RPQB, 4DEYB, 2MBHB, 2O8GI, 3SL9C, 4BXZX, 4BPE9, 4FBWC, 4A1GE, 2P58B, 3T0YB, 2GRXC, 2XG1Y, 3N7NE, 2PQNB, 2I32E, 1Z56A, 1ONVB, 2JZ3A, 2L0YB, 3ZEYR, 1XOUA, 3IYNN, 2WSCE, 4BPE0, 3QV1G, 1OEDC, 4H62K, 2F93B, 4C3ID, 4HB1A, 3S6PE, 4C0TA, 4G91A, 2NPIC, 1AYM4, 3SJHB, 1QZ7B, 1YTVM, 2XPIB, 4BKGA, 2WSCN, 3T5XB, 3FFDP, 3TVTB, 2CLYA, 2XQNA, 4C2MD, 1CQTI, 4B6HC, 3IZRb, 1YD7A, 4B4TS, 4GOWA, 2KXCB, 2WWBC, 2XB2G, 3KYSB, 1DP5B, 4BZIF, 2PRGC, 3W3WB, 2QNAB, 1H8BB, 3BTPB, 2WSCH, 4BXTA, 3IAXB, 4HQJE, 2ZNLB, 3J3Bb, 2WZLA, 3EVYA, 3TDNA, 4E05I, 1FH1A, 4KZYf, 4BYNq, 4KBBC, 3IECE, 3ZEDD, 4BQ6C, 4KIX6, 3F1IC, 3MN7S, 3UTMC, 3ABDX, 4C5HB, 1SCFA, 3M4WE, 2K3JA, 3J3Ah, 2CKZA, 1I7WB, 1UF2K, 4ACOA, 3KNDB, 2KSEA, 4AXGC, 3PMKN, 2LOXB, 3EX7D, 4I6MD, 3PVLB, 1XF5P, 4C2M2, 3E50C, 3V62C, 4HSUB, 1BYYA, 4JPBA, 4AYBQ, 4CCFA, 1H6WA, 3J3Bu, 4JHDC, 1VSZR, 4FTBD, 3QHED, 4GXBB, 2JYVA

DisProt IDs of 91 Group II Proteins:
DP00001, DP00002, DP00005, DP00006, DP00008, DP00015, DP00017, DP00024, DP00027, DP00028, DP00038, DP00039, DP00040, DP00041, DP00042, DP00057, DP00058, DP00068, DP00069, DP00070, DP00116, DP00122, DP00124, DP00128, DP00132, DP00140, DP00143, DP00145, DP00146, DP00147, DP00148_C004, DP00158, DP00163, DP00170, DP00174, DP00180_C003, DP00185, DP00186, DP00193, DP00198, DP00199, DP00205, DP00214, DP00216, DP00219, DP00222, DP00227, DP00232, DP00242, DP00253, DP00288, DP00303, DP00330, DP00332, DP00347, DP00357, DP00359, DP00367, DP00372, DP00387, DP00421, DP00441, DP00465, DP00510, DP00521, DP00531, DP00532, DP00535, DP00538, DP00540, DP00544, DP00546, DP00550, DP00560_C007, DP00563, DP00564, DP00584,

DP00586, DP00592, DP00606, DP00613, DP00626, DP00650, DP00657, DP00663, DP00664, DP00665, DP00674_C001, DP00675_C002, DP00694, DP00723
